# Feasibility, acceptability, and preliminary estimates of a multimodal AKTIVER Heart Delirium Prevention Program in patients indicated for cardiac surgery: study protocol of a randomized controlled PREVENT POstoperative Delirium (PREVENT-POD) Pilot Study

**DOI:** 10.1186/s40814-026-01923-6

**Published:** 2026-10-05

**Authors:** Julia Kühnle, Minh Tuan Dong, Joshua Heißenberg, Carlotta Derad, Elke Schettler, Claudia Neitzel, Paul T. Itting, Alexei Botnariuc, Thomas Asendorf, Birte Viehmeister, Aschraf El-Essawi, Ahmad-Fawad Jebran, Ruben Evertz, Johanna C. Badde, Carolin Steinmetz, Marcus Wuttke, Neele S. David, Juliane Spank, Daniel A. Schäfer, Ingo Kutschka, Hassina Baraki, Christoph Herrmann-Lingen, Christine Thomas, Christine von Arnim, Christopher M. Celano, Monika Sadlonova

**Affiliations:** 1 Department of Cardiothoracic and Vascular Surgery, University Medical Center Goettingen, Goettingen, Germany; 2 Department of Medical Statistics, University Medical Center Goettingen, Goettingen, Germany; 3 Department of Psychosomatic Medicine and Psychotherapy, University Medical Center Goettingen, Goettingen, Germany; 4 DZHK (German Center for Cardiovascular Research), Partner Site Lower Saxony, Goettingen, Germany; 5 Department of Clinical Pharmacy, University Medical Center Goettingen, Goettingen, Germany; 6 Department of Geriatrics, University Medical Center Goettingen, Goettingen, Germany; 7 Department of Physical Therapy, University Medical Center Goettingen, Goettingen, Germany; 8 Department of Psychiatry and Psychotherapy for the Elderly, Klinikum Stuttgart, Stuttgart, Germany; 9 Department of Psychiatry, Massachusetts General Hospital, Boston, USA; 10 Department of Psychiatry, Harvard Medical School, Boston, USA; 11 University Heart Center Basel, University Hospital Basel, Basel, Switzerland; 12 Department of Psychiatry and Psychotherapy, University Hospital of Tuebingen, Tuebingen, Germany

**Keywords:** Feasibility and acceptability, Postoperative delirium, Delirium prevention, Cardiac surgery, Older patients, Perioperative optimization, Randomized controlled trial

## Abstract

**Background:**

Postoperative delirium (POD) is a common complication in geriatric patients undergoing cardiac surgery. POD is associated with prolonged length of stay in the intensive care unit and hospital, increased mortality, institutionalization, distress, risk of cognitive impairment and dementia. The non-pharmacological, multimodal AKTIVER intervention from the multicenter PAWEL trial significantly reduced POD incidence and days with POD in older patients undergoing abdominal and orthopedic surgery. However, this positive effect on POD incidence has not yet been demonstrated in elderly patients undergoing cardiac surgery. Therefore, a specific AKTIVER Heart intervention with additional modules was developed.

**Methods:**

In the randomized controlled PREVENT-POD pilot study, 60 patients aged 65 years or older undergoing elective cardiac surgery will be recruited and 2:1 randomized in either into the AKTIVER Heart intervention group (AKTIVER Heart) or treatment-as-usual plus (TAU+) group. In total, 40 patients will receive the non-pharmacological and modified AKTIVER Heart intervention, while the other 20 will receive TAU+ with preoperative education. The primary outcome is the feasibility and acceptability of the AKTIVER Heart intervention. Secondary outcomes include the preliminary estimates of the AKTIVER Heart on POD incidence, duration and severity, cognition, quality of life, sleep, treatment- and disease expectations, distress, depression, and anxiety symptoms.

**Discussion:**

This randomized controlled pilot study aims to evaluate the feasibility and acceptability of the non-pharmacological, multimodal AKTIVER Heart intervention in elderly patients undergoing elective cardiac surgery. This trial is designed to provide evidence on acceptability and feasibility of the AKTIVER Heart, and to perform a larger, well-powered multicenter POD prevention trial in cardiac surgery.

**Trial registration:**

German Clinical Trials Register (DRKS; http://www.drks.de; DRKS00035091, Registered prospectively on November 4, 2024

**Supplementary Information:**

The online version contains supplementary material available at https://doi.org/10.1186/s40814-026-01923-6.

## Administrative information

**Table:** 

Title {1}	Feasibility, acceptability and preliminary Estimates of a Multimodal AKTIVER Heart Delirium Prevention Program in Patients Undergoing Elective Cardiac Surgery: Study Protocol of a Randomized Controlled PREVENT POstoperative Delirium (PREVENT-POD) Pilot Study
Trial registration {2a and 2b}	German Clinical Trials Register (DRK; http://www.drks.de) (DRKS00035091)
Protocol version {3}	V2.0 from October 4, 2024
Funding {4}	Faculty-Internal Research Funding for Clinical Studies, University Medical Center Goettingen, Germany
Author details {5a}	Julia Kühnle, MSc^1*^, Minh Tuan Dong, MD^1^, Joshua Heißenberg^1^, Carlotta Derad, MSc^2^, Elke Schettler^3^, Claudia Neitzel^3^, Paul T. Itting, MD^1,4^, Alexei Botnariuc, MD^1^, Thomas Asendorf, PhD^2^, Birte Viehmeister^5^, Aschraf El-Essawi, MD^1^, Ahmad-Fawad Jebran, MD^1^, Ruben Evertz, MD^1^, Johanna C. Badde^1^, Carolin Steinmetz, PhD^6^, Marcus Wuttke, PhD^7^, Neele S. David^7^, Juliane Spank, PhD^8^, Daniel A. Schäfer, MD^9,10^, Ingo Kutschka, MD^,,11^, Hassina Baraki, MD^11^, Christoph Herrmann-Lingen, MD^3,4^, Christine Thomas, MD^8,12^, Christine von Arnim, MD^4,6^, Christopher M. Celano, MD^9,10^, Monika Sadlonova, MD^1,3,4,6,9^ ^1^Department of Cardiothoracic and Vascular Surgery, University Medical Center Goettingen, Goettingen, Germany ^2^Department of Medical Statistics, University Medical Center Goettingen, Goettingen, Germany ^3^Department of Psychosomatic Medicine and Psychotherapy, University Medical Center Goettingen, Goettingen, Germany ^4^DZHK (German Center for Cardiovascular Research), partner site Lower Saxony, Germany ^5^Department of Clinical Pharmacy, University Medical Center Goettingen, Goettingen, Germany ^6^Department of Geriatrics, University Medical Center Goettingen, Goettingen, Germany ^7^Department of Physical Therapy, University Medical Center Goettingen, Goettingen, Germany ^8^Department of Psychiatry and Psychotherapy for the Elderly, Klinikum Stuttgart, Stuttgart, Germany ^9^Department of Psychiatry, Massachusetts General Hospital, Boston, USA ^10^Department of Psychiatry, Harvard Medical School, Boston, USA ^11^University Heart Center Basel, University Hospital Basel, Basel, Switzerland ^12^Department of Psychiatry and Psychotherapy, University Hospital of Tuebingen, Tuebingen, Germany
Name and contact information for the trial sponsor {5b}	University Medical Center Goettingen, Georg-August-University, Robert-Koch-Street 40, 37075 Goettingen, Germany
Role of sponsor {5c}	The sponsor has no role in the design of the study, data collection, data analysis, interpretation of data, or writing of the manuscript

## Introduction

### Background and rationale

In Germany, 23.2% of the total population (19.5 million) are aged 65 years or older. By 2050, the number of people over 65 is expected to increase to 22.4 million [1]. As part of this sociodemographic change, degenerative cardiovascular diseases, such as coronary artery disease and aortic stenosis requiring surgical procedures (e.g., coronary artery bypass grafting or aortic valve replacement), are expected to increase in incidence [2]. Postoperative delirium (POD), characterized by fluctuations in consciousness, cognition, and psychomotor function [3], is a common complication following cardiac surgery, affecting up to 55% of patients undergoing these procedures [4–9]. Older, multimorbid, and frail patients are at particularly high risk of developing POD [10–12].

In addition to acute symptoms of POD, the development of POD can have functional, medical, cognitive, and psychological consequences. POD has been associated with increased morbidity, mortality, prolonged length of stay (LOS) in hospital and in the intensive care unit (ICU) [6, 11, 13, 14], higher readmission rates [15, 16], and greater prevalence of falls [17]. Furthermore, patients who experienced POD may suffer from long-term cognitive impairment and have up to a sixfold increased risk of developing dementia [11, 13–15, 18]. Additionally, POD with hyperactive and/or psychotic symptoms (e.g., agitation, delusions, visual hallucinations, and disorientation) often leads to posttraumatic stress disorder (PTSD), anxiety disorders, or depression [11]. Finally, POD can significantly increase healthcare costs, leading to up to $64,000 in healthcare expenses per patient per year [19]. This highlights the importance of POD prevention in older patients undergoing cardiac surgery [19, 20].

Multi-component programs to prevent delirium have shown promise in many surgical populations. These interventions can significantly reduce delirium incidence by up to 40% [21–24]. Additionally, delirium prevention programs have been shown to decrease delirium severity and the incidence of falls [23].

In the multicenter, stepped-wedge cluster randomized PAWEL study [25], the effectiveness of a multimodal, non-pharmacological POD prevention program named “Alltags- und KognitionsTraining und Interdisziplinarität Verbessert das Ergebnis und mindert das Risiko” (“everyday skills and cognition training and interdisciplinarity improves outcome and mitigates risk”) (AKTIVER [“more active”]) was investigated in 1470 patients aged ≥70 years undergoing elective orthopedic, visceral, or cardiac surgery. The AKTIVER intervention resulted in a significant reduction in both the incidence of POD and the number of POD days in patients undergoing orthopedic and visceral surgery (odds ratio [OR] 0.59; 95% CI 0.35–0.99, *p* = 0.047). In contrast, AKTIVER did not significantly impact POD incidence in patients undergoing cardiac surgery (OR 1.18; 95% CI 0.70–1.99, *p* = 0.54) [22]. However, AKTIVER led to a significant reduction in LOS for cardiac surgery patients [22].

The lack of effectiveness of the AKTIVER intervention in cardiac surgery highlighted the need for a modification of the AKTIVER, to target those factors most strongly associated with POD in this population [4, 5, 8, 26, 27]. The newly introduced preoperative, intraoperative, and postoperative components of AKTIVER Heart are grounded in established and emerging risk factors for postoperative delirium (POD) following cardiac surgery and target potentially modifiable mechanisms across the perioperative trajectory. Preoperatively, several patient-related factors have consistently been associated with an increased risk of POD, including anxiety, depressive symptoms [28], frailty [29], sarcopenia, malnutrition [30], and a high anticholinergic medication burden [31]. Frailty and sarcopenia may reduce resilience to surgical stress, whereas malnutrition and iron deficiency are associated with diminished physiological reserve and impaired postoperative recovery. In addition, polypharmacy and exposure to anticholinergic medications may contribute to cognitive dysfunction and delirium susceptibility. To address these risk factors, AKTIVER Heart implements multimodal prehabilitation [29, 32–34] and perioperative strategies [35], including psychoeducation, psychological preparation, medication review and optimization, nutritional support, correction of iron deficiency, and interventions targeting frailty and physical deconditioning. Collectively, these measures aim to enhance both physiological and psychological resilience before surgery and thereby reduce vulnerability to POD. Intraoperatively, procedure-related factors such as prolonged cardiopulmonary bypass (CPB) duration, extended ischemic time, complex or combined surgical procedures, and intraoperative hemodynamic instability have been associated with increased delirium risk [26, 36, 37]. Consequently, AKTIVER Heart emphasizes optimized surgical and anesthetic management, including minimization of CPB and ischemic times whenever feasible, optimized volume management, standardized analgesic protocols, and reduction of potentially delirium-promoting exposures [35, 38]. Postoperatively, several potentially modifiable factors contribute to POD development, including prolonged mechanical ventilation, inadequate pain control [39], sleep disruption, immobilization, exposure to deliriogenic medications (e.g., benzodiazepines) [40], and prolonged use of drains and catheters [37, 41]. Accordingly, AKTIVER Heart integrates evidence-based postoperative strategies such as early extubation, opioid-sparing analgesia, avoidance of benzodiazepines whenever possible, promotion of sleep and orientation, early oral feeding, timely removal of drains and catheters, and structured mobilization [30, 35, 38, 42]. In addition, AKTIVER Heart includes components aimed at reducing environmental stressors and strengthening patient resources are intended to support cognitive recovery and resilience during the vulnerable postoperative period. Taken together, AKTIVER Heart extends the original AKTIVER program through a multimodal, biopsychosocial, and interprofessional approach that addresses modifiable risk factors across the entire perioperative pathway. By targeting psychological, biological, behavioral, and treatment-related mechanisms implicated in POD, the intervention aims to enhance perioperative resilience, reduce POD risk, and improve postoperative recovery following cardiac surgery. Building on our previous work and emerging evidence on POD prevention, we developed AKTIVER Heart as a multimodal, non-pharmacological, and interprofessional intervention tailored to the needs of older cardiac surgery patients. In the randomized controlled PREVENT-POD pilot trial, we aim to evaluate the feasibility, acceptability, and preliminary estimates of the AKTIVER Heart intervention in 60 adults aged ≥65 years undergoing elective cardiac surgery.

### Objectives

The primary objective of the randomized controlled PREVENT-POD pilot study is to evaluate whether the multimodal, non-pharmacological AKTIVER Heart intervention is feasible and well accepted among patients aged ≥65 years undergoing elective cardiac surgery. A further primary objective is to assess the feasibility of the overall study design of the randomized controlled PREVENT-POD pilot study.

The secondary objective is to explore the preliminary estimates of the AKTIVER Heart intervention compared with treatment as usual including preoperative education (TAU+). Outcomes include POD incidence and severity, use of antipsychotic drugs, anticholinergic burden (ACB), quality of life (QoL), frailty, cognitive function, depression, anxiety, sleep quality, treatment- and disease-related expectations, complications, LOS in the hospital and ICU, assessed at discharge and at the 3-month follow-up (FU) visit.

### Trial design

PREVENT-POD is a randomized controlled pilot trial in patients aged ≥65 years undergoing elective cardiac surgery. The primary aim of PREVENT-POD is to investigate the acceptability and feasibility of the AKTIVER Heart intervention and the feasibility of the PREVENT-POD study design. The secondary aim is to analyze the preliminary estimates of the AKTIVER Heart intervention compared to TAU+ at discharge and 3-month FU. Preliminary estimates outcomes include delirium incidence, duration and severity, cognition, quality of life, sleep, treatment- and disease expectations, distress, depression, and anxiety symptoms. The study assessors will be blinded to randomization for the baseline assessments. Ethical approval for this study protocol was obtained from the Ethics Committee of the University Medical Center Goettingen on October 4, 2024 (#15/9/24). The PREVENT-POD study was prospectively registered in the German Clinical Trials Register (DRKS; https://www.drks.de) (DRKS00035091) on November 4, 2024.

## Methods: participants, interventions and outcomes

### Study setting

In total, 60 patients aged ≥65 years undergoing elective cardiac surgery will be recruited in the Department of Cardiac, Thoracic and Vascular Surgery of the University Medical Center Goettingen, Germany. Inclusion and exclusion criteria are summarized in Table 1. All patients aged ≥65 years who are already scheduled for an elective cardiac surgery will be screened for eligibility and contacted by a study nurse via telephone to inquire whether they are interested in participating in the PREVENT-POD trial. After initial screening, the identified patients will be thoroughly informed about the planned research project. All patients will receive study information, and a consent form via email or postal mail. If they express interest in participating in the study and have sufficient time to consider their decision, patients will be invited to the baseline visit (t0) within a timeframe of 2 days to 3 weeks prior to their cardiac surgery. Informed consent will be collected by study physician after providing detailed study information and prior to baseline assessment. All study participants will receive a signed copy of informed consent form. Patients may withdraw their consent to participate in the study at any time.

**Table 1:** Inclusion and exclusion criteria of the PREVENT-POD study

Inclusion criteria	1. Patients aged ≥65 years 2. Elective cardiac surgery (with or without cardiopulmonary bypass, with or without sternotomy, duration of surgery >60 min) 3. Ability to give study consent 4. Willingness to participate voluntarily after informed consent with signed consent form 5. Ability to speak, read and understand German language
Exclusion criteria	1. Unable to provide written study consent 2. Severe dementia, severe mental disorders (i.e., acute psychosis, severe depressive episode, acute suicidal ideation) or acute delirium 3. Diagnosis of ongoing alcohol and/or drug use disorder 4. Unstable angina pectoris, STEMI or NSTEMI within the last 2 weeks 5. Heart failure, NYHA IV 6. Severe cardiac arrhythmia refractory to therapy 7. Advanced (metastasized) oncological disease 8. Participation in another intervention study (participation in registry studies is permitted)

*NSTEMI* non-ST elevation myocardial infarction, *NYHA* New York Heart Failure Association, *STEMI *ST elevation myocardial infarction

**Fig. 1:**
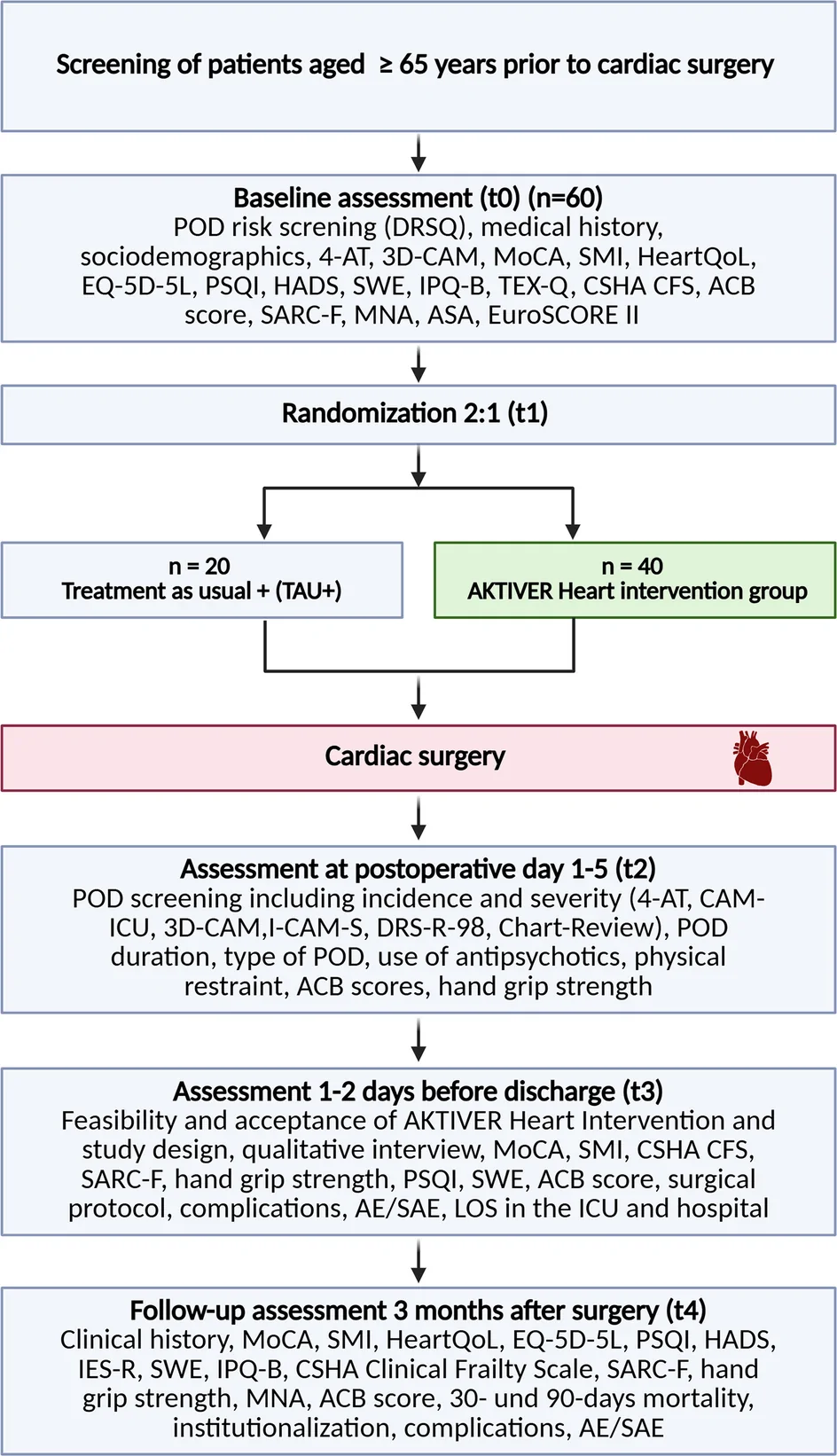
Flow diagram of the PREVENT-POD study *3D-CAM* 3-Dimension-Confusion Assessment Method, *4-AT* 4-As-Test, *ACB* *Score* Anticholinergic Burden, *AE/SAE* Adverse Events/Serious Adverse Events, *ASA* American Society of Anethesiologist, *CAM-ICU* Confusion Assessment Method-Intensive Care Unit, *I-CAM-S* ICD-10-Confusion Assessment Method-Severity, *CSHA* Canadian Study of Health and Aging, *CFS* Clinical Frailty Scale, *DRS-R-98* Delirium Rating Scale Revised, *DRSQ* Delirium Risk Screening Questionnaire, *EQ-5D-5L* five-dimensional European Quality of life Questionnaire, *EUROScore II* European System for Cardiac Operative Risk Evaluation, *HADS* Hospital Anxiety and Depression Scale, *HeartQoL* Heart-related Quality of Life, *ICU* Intensive Care Unit, *IES-R* Impact of Event Scale-Revised, *LOS* Length of stay, *IPQ-B* Illness Perception Questionnaire, *MNA* Mini Nutritional Assessment, *MoCA* Montreal Cognitive Assessment, *POD* Postoperative Delirium, *PSQI* Pittsburgh Sleep Quality Index, *QoL* Quality of Life, *SARC-F* Strength, Assistance with walking, Rise from a chair, Climb stairs and Falls, *SMI* subjective memory impairment, *SWE* Self Efficacy Expectations, *TAU* Treatment as Usual, *TEX-Q* Treatment Expectation Questionnaire

The data collection is distributed across four visits. The first visit is the preoperative screening containing medical history, blood samples, POD risk screening and assessments of secondary outcomes (t0). After finishing the baseline study assessments, patients will be randomized into either AKTIVER Heart intervention or TAU+ group and will be informed about the result of randomization (t1). Patients in the AKTIVER Heart group will receive initial preoperative optimization, and patients in the TAU+ group will receive preoperative education. POD symptoms will be assessed during the first five postoperative days twice daily, and patients in the AKTIVER Heart group will additionally receive non-pharmacological POD prevention interventions (t2). At discharge, feasibility and acceptability will be documented and assessments of cognition, sleep quality and frailty will be conducted (t3). We will perform a FU visit 3 months after surgery (t4) (Fig. 1 and Table 2). The personnel involved in data collection will undergo comprehensive training to standardize procedures and minimize variability. This includes instruction on administering assessments and recording data accurately.

**Table 2:** Study visits and assessments

Assessment	Screening and preoperative assessments			Postoperative assessments		
**Visit**		**t0/t1**		**t2**	**t3**	**t4**
**Time**	**Screening**	**Baseline/Randomization**		**Postoperative days 1–5 (longer with POD)**	**1–2 days before discharge**	**3 months after surgery**
Inclusion and exclusion criteria	**x**		Elective cardiac surgery			
Study information and consent form		**x**				
DRSQ (POD risk)		**x**				
ACB score (anticholinergic burden)		**x**		**x**	**x**	**x**
EuroSCORE II and ASA		**x**				
Examinations (e.g., duplex sonography, CT, ECG)		**x**		**x**	**x**	
Laboratory measurements		**x**		**x**	**x**	
Medical history, sociodemographic and medication		**x**				**x**
MoCA (cognitive performance)		**x**			**x**	**x**
SMI (subjective cognitive impairment)		**x**			**x**	**x**
HeartQoL (heart-related QoL)		**x**				**x**
EQ-5D-5L (generic QoL)		**x**				**x**
PSQI (sleep quality)		**x**			**x**	**x**
HADS (anxiety and depression)		**x**				**x**
IES-R (post-traumatic stress symptoms)						**x**
SWE (self-efficacy)		**x**			**x**	**x**
IPQ-B (illness perception)		**x**				**x**
TEX-Q (treatment expectation)		**x**				
CSHA Clinical Frailty Scale (frailty)		**x**			**x**	**x**
SARC-F (sarcopenia)		**x**			**x**	**x**
Hand grip strength (frailty)		**x**		**x**	**x**	**x**
MNA (malnutrition)		**x**		**x**		**x**
CAM-ICU/3D-CAM/4-AT (POD incidence)		**x**		**x**		
CAM-S/DRS-R-98 (POD severity)				**x**		
POD duration (if applicable)				**x**		
Type of POD (if applicable)				**x**		
Use of antipsychotics				**x**	**x**	
Use of physical restraint				**x**	**x**	
Peri- und postoperative complications					**x**	**x**
Length of stay (ICU/hospital)					**x**	
Feasibility of the AKTIVER Heart intervention					**x**	
Acceptance of the AKTIVER Heart intervention					**x**	
Qualitative interview of AKTIVER Heart patients					**x**	
Surgical protocol					**x**	
Institutionalization/need for care						**x**
30- and 90-day-mortality						**x**
AE/SAE				**x**	**x**	**x**

*3D-CAM* 3-Dimension Confusion Assessment Method, *4-AT *4-As-Test, *ACB* Anticholinergic Burden, *AE/SAE* Adverse Events/Serious Adverse Event, *ASA* American Society of Anesthesiologist, *CAM-ICU* Confusion Assessment Method-Intensive Care Unit, *CAM-S* Confusion Assessment Method-Severity, *CSHA Clinical Frailty Scale* Canadian Study of Health and Aging Clinical Frailty Scale, *CT* Computertomography, *DRS-R-98 *Delirium Rating Scale Revised, *DRSQ *Delirium Risk Screening Questionnaire, *ECG *Electrocardiogram, *EQ-5D-5 *Five-Dimensional European Quality of Life Questionnaire, *EUROScore II *European System for Cardiac Operative Risk Evaluation, *HADS *Hospital Anxiety and Depression Scale, *HeartQoL* Heart-related Quality of Life, *ICU* Intensive Care Unit, *IES-R *Impact of Event Scale-Revised, *IPQ-B *Illness Perception Questionnaire, *MNA *Mini Nutritional Assessment, *MoCA *Montreal Cognitive Assessment, *POD* Postoperative Delirium, *PSQI *Pittsburgh Sleep Quality Index, *QoL *Quality of Life, *SARC-F *Strength, Assistance with walking, Rise from a chair, Climb stairs and Falls, *SMI *Subjective Memory Impairment, *SWE *Self Efficacy Expectations, *TEX-Q *Treatment Expectation Questionnaire

#### Baseline assessment (t0)

Initially, the individual POD risk will be assessed using the Delirium Risk Screening Questionnaire (DRSQ) [9, 43]. This tool was originally developed in context of the PAWEL study [25]. The DRSQ consists of several categories that account for potential POD risk. The first part of the DRSQ is the geriatric check [44, 45], which includes five items: mobility, independence in activities of daily living (ADLs), cognition, mental health, and previous hospitalizations. Patients who are impaired in at least two of these items are classified as geriatric patients. The second part of the DRSQ involves a cognitive screening test, the 6-Item Cognitive Impairment Test (6-CIT), which assesses orientation, short-term memory, and attention (counting backward from 20 to 1 and listing the months of the year backwards) [46–48]. The total score ranges from 0 to 28 points, with 0–7 indicating normal cognition, 8–9 suggesting mild cognitive impairment, and 10–28 indicating significant cognitive impairment. The third part of the DRSQ collects general patient information (19 items), including: age > 70 years, laboratory parameters (creatinine > 1.00 mg/dl; C-reactive protein > 5 mg/l; hemoglobin < 11.5 g/dl; protein < 6.6 g/dl; electrolytes outside the normal range), an American Society of Anesthesiologist (ASA) score ≥ 3, daily medication intake (more than six medications) and use of medications with a POD-inducing potential (ACB score ≥ 1). The ACB score assesses anticholinergic load and should be particularly considered in geriatric and multimorbid patients [49]. The score is calculated based on a list of medications commonly used in Germany [49]. A medication with no anticholinergic effect has an ACB score of 0, a mild anticholinergic effect is assigned a score of 1, a moderate effect a score of 2, and a strong effect a score of 3. The total ACB score is the sum of individual medication scores. If the total score is ≥3, it is recommended to reduce the ACB by discontinuing or replacing medications if possible [49]. Further steps in the DRSQ include recording preoperative diagnoses of depression, stroke, or dementia, German care dependency level, and whether the patient resides in a nursing home [50]. Participants are also asked about previous episodes of delirium and any recent incidence of falls. Additionally, self-reported subjective memory impairment (SMI), alcohol consumption, and smoking status are documented [50]. Finally, hand grip strength is measured using the Jamar® Hydraulic Hand Dynamometer. POD risk is measured by summing the relevant DRSQ items. Based on the DRSQ scoring guidelines, a score of <2 requires no further action, 2–4 indicates the need for further POD risk assessment, and >4 suggests initiating preventive measures.

Preoperative cognitive function will be assessed using the Montreal Cognitive Assessment (MoCA) [51]. The MoCA is a validated cognitive screening tool comprising 30 items, specifically designed to detect mild cognitive impairment and early stages of dementia [51]. The test evaluates multiple cognitive domains, including short-term memory, visuospatial abilities, attention, concentration, working memory, language, and temporal and spatial orientation. The maximum possible score is 30, with a total score of ≤26 indicating cognitive impairment [51–53].

Heart-related QoL is assessed using the HeartQoL questionnaire [54, 55]. The HeartQoL is a 14-item self-report instrument designed to measure health-related QoL, which is reliable, valid, and sensitive to changes across various cardiac populations [54]. In our study, we will use the German version of the questionnaire [55]. Overall QoL will be measured using the five-dimensional EuroQoL questionnaire (EQ-5D-5L) [56]. The EQ-5D-5L covers five dimensions: mobility, self-care, usual activities, pain/discomfort, and anxiety/depression. Each dimension is rated on a 5-point scale. The responses to the individual items are combined into a quality-of-life index using established algorithms. Additionally, patients assess their health status on a visual analog scale (EQ-VAS; range 0–100) [35].

Sleep quality will be assessed using the Pittsburgh Sleep Quality Index (PSQI), a self-report questionnaire designed to evaluate sleep quality in clinical settings [57–59]. In addition to self-assessment, the PSQI includes five questions for external evaluation, though these are not included in the quantitative analysis. The questionnaire consists of seven components: subjective sleep quality, sleep latency, sleep duration, sleep efficiency, sleep disturbances, use of sleep medication, and daytime dysfunction. These components refer to the subjective sleep quality over the past 4 weeks. The total score ranges from 0 to 21, with a score >5 indicating impaired sleep quality [58].

Anxiety and depression symptoms will be measured using the Hospital Anxiety and Depression Scale (HADS) [60, 61]. The HADS measures the severity of anxiety and depression in physically ill patients. It consists of 14 self-assessment items, rated on a four-point Likert scale, whereas higher scores indicate greater severity of symptoms [60, 61]. Self-efficacy will be assessed using the General Self-Efficacy Scale [62]. This scale consists of 10 self-report items that evaluate an individual’s perceived effectiveness in finding solutions and managing difficulties and challenges [62].

Treatment expectations will be assessed using the Treatment Expectation Questionnaire (TEX-Q) [63]. The short version of the TEX-Q consists of 15 items and uses a ten-point scale to measure patients’ expectations regarding their heart surgery. Disease-related expectations will be measured using the short version of the Illness Perception Questionnaire (IPQ-B) [64]. The short version consists of eight items related to general health, rated on a scale from 0 to 10, and a prompt to list the most important subjective reasons for the perceived cause of the disease.

Frailty will be measured using the German version of the Canadian Study of Health and Aging (CSHA) Clinical Frailty Scale (CFS) [65, 66]. This scale consists of 9 categories, taking into account physical fitness, disease symptoms, daily activities, and cognition. A score of 1 describes a very fit person, while a score of 9 represents someone who is extremely ill. A score of 5 or higher indicates frailty [66]. Sarcopenia will be assessed using the Strength, Assistance with Walking, Rise from a Chair, Climb Stairs and Falls (SARC-F) questionnaire [67]. Based on five items, which are already included in the name, patients rate each category from “not severe” (0 points) to “very severe” (2 points), or “no falls” (0 points) to “4 or more falls” (2 points). A maximum of 10 points can be achieved, with a score of ≥4 indicating sarcopenia [67, 68].

Nutritional status will be assessed using the Mini Nutritional Assessment—Short Form (MNA-SF) [69]. The MNA-SF includes the following evaluation criteria: food intake and weight loss over the last 3 months, mobility, acute illness and psychological stress, neuropsychological issues, and body weight. For each category, up to three points can be scored. A score of 12–14 points indicates a normal nutritional status, 8–11 points suggests a risk of malnutrition, and 0–7 points indicate the presence of malnutrition [69].

Additionally, we will assess whether delirium is already present in study participants prior to surgery. All delirium assessments will be performed by trained study personnel. Assessors will receive standardized training in the administration and scoring of the CAM-ICU, 3D-CAM, and 4AT, as well as instruction regarding ICD-10 and DSM-5 diagnostic criteria for delirium. The training procedures are based on our previous FINDERI study [9, 43], in which structured delirium training and regular supervision were successfully implemented to ensure standardized delirium assessments. Throughout the study, assessors will participate in regular supervision meetings and case discussions with experienced clinicians to promote adherence to assessment procedures and ensure consistent application of delirium diagnostic criteria. Delirium will be assessed using the modified Confusion Assessment Method (CAM), the 3D-CAM [70, 71], as well as the quick 4-A Test [4-AT] [72] widely recommended by several guidelines. The 3D-CAM consists of three assessment categories: questions to the patients, observer ratings and two optional items [73]. In the first category, seven questions assess thinking and memory, and the patient provides a subjective assessment of their current mental state, orientation, and presence of hallucinations. Answers in this category are marked as true or false, or yes or no for subjective questions [73]. The next category consists of 10 observational criteria, which are also answered yes or no. Each question and task in the 3D-CAM corresponds to one of the four CAM-algorithm items. If item 1 and 2 and either 3 or 4 are present, the screening is positive for delirium. The two optional items on acute change are only required if characteristic 1 is absent but characteristics 2 and either 3 or 4 are present. The 4AT consists of four items [72]. The first item assesses the level of alertness. The next two items involve the Abbreviated Mental Test-4 (AMT-4) [74] and the evaluation of attention by having the patients recite the months of the year backwards. Item 4 assesses whether there is an acute change in mental state or fluctuation of symptoms. The 4AT can score a total of 12 points, which are evaluated as follows: 0 points = no delirium or cognitive impairment; 1–3 points = possible cognitive impairment; and ≥4 points = possible delirium [72].

Finally, a detailed clinical history and sociodemographic data will be collected. The baseline visit will approximately take one and a half hours.

#### Randomization (t1)

Randomization will be performed utilizing the randomization module of the Research Electronic Data Capture (RedCap) Database (Vanderbilt University). The allocation sequence, using permuted blocks of varying lengths, was generated by a statistician of the Department of Medical Statistics. Randomization will be conducted in a 2:1 ratio, with 40 patients allocated to the AKTIVER Heart intervention group and 20 patients to the TAU+ group. This allocation ratio was chosen because the primary feasibility (intervention and study design) and acceptability endpoints are assessed within the intervention group only. A larger intervention group increases the precision of these estimates and provides more detailed information on implementation processes to inform the design of a future definitive trial. As this is a feasibility study rather than an efficacy trial, this was considered preferable despite the reduced efficiency for between-group comparisons. The trial remains two-armed as secondary endpoints compare outcomes between the AKTIVER Heart intervention and the TAU+. Every patient will be allocated to one of the groups by one of the clinical investigators and will be informed about the results of randomization.

#### Postoperative visits for POD and hand grip screening (t2)

Delirium diagnosis for the preliminary estimates will be determined according to the CAM algorithm using the CAM-ICU [75] in the ICU and 3D-CAM [70] in general ward. Patients will be screened for POD during the first five postoperative days twice daily (in the morning between 8 and 10 A.M. and evening between 4 and 6 P.M.). The 4-AT [72] will additionally be administered at each assessment time point and will be used for secondary analyses, including comparison of POD incidence and diagnostic performance across different delirium screening instruments. POD severity will be assessed twice daily using two instruments: the ICD-10-Confusion Assessment Method-Severity (I-CAM-S) and the Delirium Rating Scale-Revised-98 (DRS-R-98) [76, 77]. The I-CAM is an operationalized German version of the CAM Short Form [78], which was extended and operationalized to assess delirium severity in analogy to the CAM Severity Short Form Score [76]. The I-CAM-S version was refined and validated as part of the PAWEL study [22]. CAM-Algorithm Items are rated as absent (0) or present (1), items 2 to 4 are additionally rated as mild (1) or severe (2), resulting in a severity range of 0 to 7 points [76]. The DRS-R-98 comprises a total of 16 items, including 13 severity items and 3 diagnostic items for delirium [77]. The severity items are rated on a scale from 0 to 3, while the diagnostic items are rated on a scale of either 0–2 or 0–3. The maximum possible score for the severity items is 39, and the overall maximum score is 46. Higher scores indicate more severe delirium, whereas a score of 0 indicates the absence of delirium [77]. Hand grip strength will be measured once daily during the morning visit. In the event of POD occurrence, patients will continue to be screened until the POD subsides, and the duration of POD will be documented. Since POD symptoms often manifest at night, a chart review will also be conducted. Additionally, electronic documentation will be reviewed, focusing on: (a) POD symptoms during the night, (b) use of antipsychotic drugs or dexmedetomidine, (c) use of physical restraints. This assessment will take approximately 5 to 10 min per patient per data collection.

#### Postoperative visit before discharge (t3)

In the AKTIVER Heart group, feasibility and acceptability will be assessed approximately 1 to 3 days before discharge. Feasibility will evaluate whether patients received preoperative education on perioperative procedures and POD as well as perioperative somatic and psychological preparation and at least one AKTIVER Heart module per day. Acceptability will be assessed using a structured interview and the evaluation of various aspects on numerical scales from 0 to 10. Additionally, standardized surgical protocol, intra- and postoperative complications, LOS in the ICU and hospital will also be documented in both study groups. Finally, assessments will be conducted for cognition (MoCA, SMI), sleep quality (PSQI), self-efficacy (SWE), handgrip strength, and sarcopenia (SARC-F) in both study groups. This visit will approximately take 45 min, up to 1 h.

#### Follow-up visit 3 months after surgery (t4)

Three months after surgery, all study participants will be invited to the hospital for a FU visit. During the FU visit, new information on preexisting conditions and sociodemographic data will be collected. Symptoms of PTSD will be assessed using the Impact of Event Scale Revised (IES-R) [79, 80]. The IES-R measures PTSD symptoms (i.e., intrusion, avoidance and hyperarousal) caused by traumatic events (i.e., cardiac surgery) [80]. The IES-R consists of 22 items on a Likert scale ranging from 0 (not at all) to 5 (often) [80]. The other assessments are identical to those conducted during the baseline visit (t0) without laboratory measurements. This FU visit will approximately take 1 h.

#### AKTIVER Heart intervention

AKTIVER Heart intervention delivery requires an interprofessional team consisting of cardiac surgeons, nurses, a psychosomatic expert, physiotherapists, pharmacists, and trained study personnel. All intervention staff undergo a standardized 2-day training conducted by experienced AKTIVER trainers before study initiation. To ensure consistent intervention delivery, dedicated study personnel coordinate and perform the AKTIVER Heart intervention and document intervention adherence using standardized procedures. Based on preliminary implementation estimates, intervention delivery, documentation, and patient contact require approximately 60 min per patient per day, allowing a maximum of four patients to be managed daily by the intervention team. One objective of this pilot trial is to evaluate the practical integration of AKTIVER Heart into routine perioperative cardiac surgical care. Accordingly, feasibility outcomes will include intervention delivery, adherence, staffing requirements, resource utilization, workflow integration, and barriers to implementation. These data will inform the refinement of intervention procedures and the planning of a future multicenter randomized controlled trial.

**Fig. 2:**
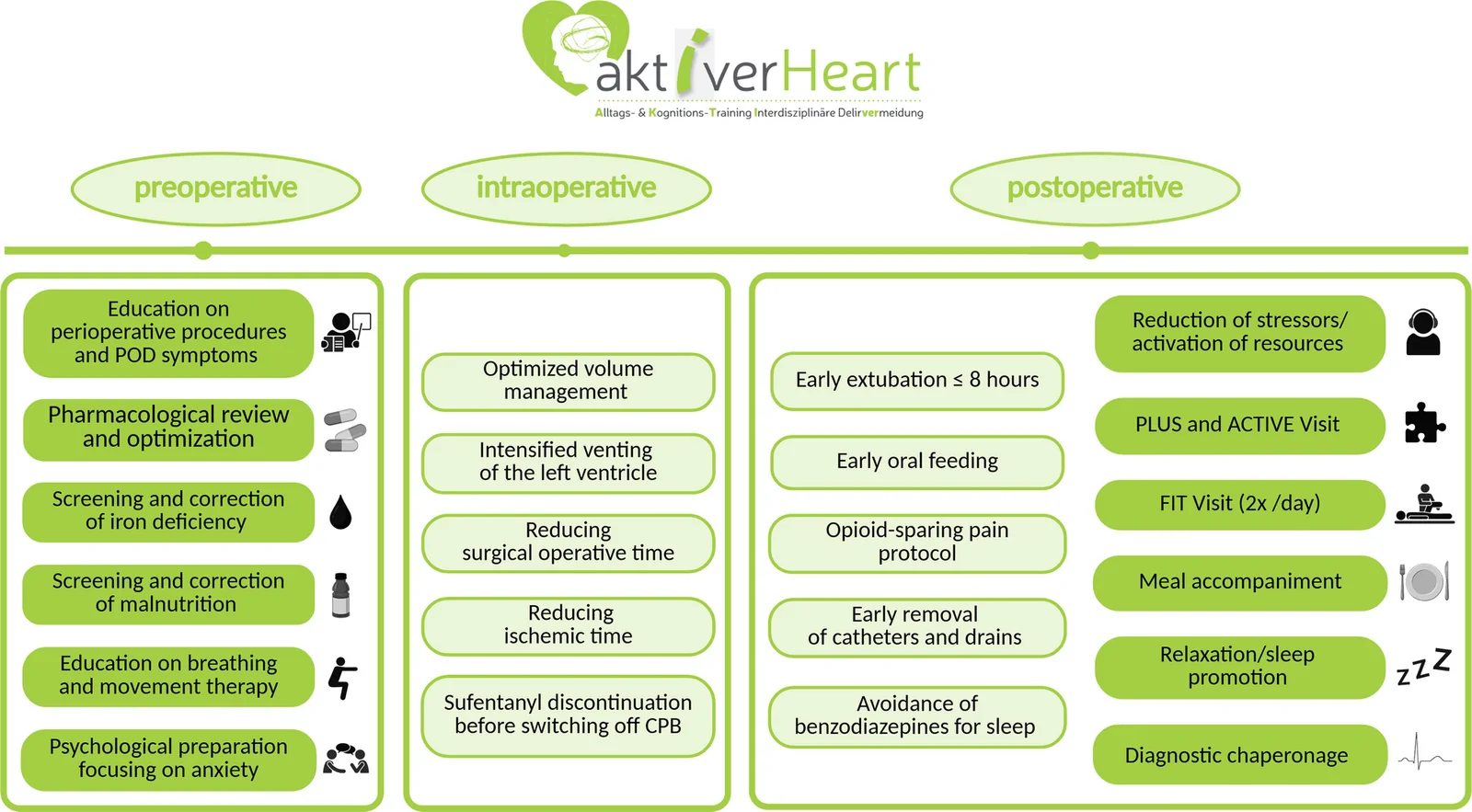
Components of the AKTIVER Heart intervention *CPB* Cardiopulmonary bypass, *LV* Left Ventricular, *POD* Postoperative Delirium

Initially, patients in the intervention group will receive a detailed explanation of the AKTIVER Heart intervention. The first step of AKTIVER Heart is the module “perioperative optimization.” The “perioperative optimization module” is one of the new additions to the original AKTIVER intervention from the PAWEL study [22]. The module has been designed in alignment with the perioperative principles of Enhanced Recovery After Surgery (ERAS) [81] and aspects of the Safe Brain Initiative (SBI) [30, 42]. It addresses a range of predisposing and precipitating delirium risk factors like anxiety [28], frailty [29], sarcopenia, malnutrition [30] mechanical ventilation time in the ICU, and volume load during surgery [26, 35–41]. This module consists of the following interventions: (a) pharmacological review and if necessary optimization of medication, (b) education about perioperative procedures and POD symptoms (identical to the TAU+ group), (c) preoperative exercises and breathing therapy [81–84], and (d) interdisciplinary optimization of intraoperative/perioperative treatment. Patients are encouraged to implement breathing and movement therapy at home or in the hospital before the surgery. The movement therapy consists of light endurance training in the form of walks three times per week for 10–60 min, as well as various strength exercises that can be performed daily while seated. To strengthen the respiratory muscles and develop breathing awareness, breathing therapy should also be practiced with an incentive spirometer before surgery. Intraoperative optimization includes optimizing volume management, intensifying venting of the left ventricle, reducing ischemic and surgical time, and discontinuing of sufentanyl before the cardiopulmonary bypass is switched off [26, 35–41]. Postoperative optimization includes extubating patients within 8 h, if possible, allowing them to eat and drink as soon as possible [38, 82] and minimizing the use of benzodiazepines and opioids for sedation [39]. An interprofessional team ensures that all medications taken preoperatively, especially antidepressants, are continued postoperatively. It is also ensured that catheters and drains are removed at the earliest possible stage to reduce the risk of infection and promote independence [38]. Another addition to AKTIVER is the reduction of preoperative distress or anxiety. Therefore, patients in the AKTIVER Heart group will be psychologically prepared with a focus on anxiety reduction, optimization of treatment expectations, and education [28, 85]. The AKTIVER Heart interventions include, in addition to the optimization of perioperative and psychological management described before, the modules [22] presented in Fig. 2 and Table 3.

**Table 3:** AKTIVER heart delirium prevention program

AKTIVER HEART Module	Content	Delirium risk factors addressed	Main indications	Model
Perioperative Optimization	**Preoperative:** education on perioperative procedures and POD symptoms, pharmacological review, screening and correction of iron deficiency, screening and correction of malnutrition, education on breathing and movement therapy, psychological preparation **Intraoperative:** optimized volume management, intensified venting of the left ventricle, reducing surgical operative time, reducing ischemic time, sufentanyl discontinuation before switching off CPB **Postoperative:** early extubation, early oral feeding, opioid-sparing pain protocol, early removal of catheters and drains, avoidance of benzodiazepines for sleep	Anxiety, depressive symptoms [28], frailty [29], sarcopenia, malnutrition [30], high anticholinergic medication burden [31], mechanical ventilation time in ICU, volume load during surgery, length of stay in ICU and length of surgery, use of deliriogenic medication [26, 35–41]	Modification of risk factors for POD and potentially modifiable mechanisms during the perioperative period	ERAS [38, 81–83] SBI [30, 42]
Reduction of stressors and activation of resources	Favorite music, noise cancelling headphones, MP3 players, identify personal resources and preferences with the “life flower”-tool, VR-guided relaxation	Stress, anxiety [28]	Reduction of stressors and activation of resources	AKTIVER [22], CHOPS [86, 87], HELP [88], help+ [89], SBI [30, 42]
PLUS and Active visit	Reorientation covering time, location, situation and person (e.g., using clocks, calendars, hearing aids and glasses), verbal information transfer, cognition promotion (e.g., VR-related activation, quiz, games)	Cognitive dysfunction, hearing and vision impairment [21], stress [28]	Cognitive activation and Reorientation	AKTIVER [22], CHOPs [86, 87], HELP [88], help+ [89], SBI [30, 42]
FIT Visit	Exercises led by a physical therapist twice daily e.g. mobilization, movement exercises in bed, breathing therapy	Immobilization, frailty [29], sarcopenia [30]	Mobilization and prevention of e.g. pneumonia, decubitus, muscle loss	AKTIVER [22], HELP [88], help+ [89], mHELP [90]
Meal accompaniment	Assistance with meals and eating, stimulating appetite	Malnutrition [30]	Nutrition, fluid intake	AKTIVER [22], HELP [88], mHELP [90], help+ [89]
Promotion of sleep/relaxation	Relaxing music, aroma therapy, sleeping tea, sleeping mask, noise cancelling headphones, calming conversations	Anxiety, stress [28], sleep disturbance [89]	Sleep promotion and relaxation, sleep–wake rhythm	AKTIVER [22], HELP [88], help+ [89], SBI [30, 42]
Diagnostic chaperonage	Accompanying patients to examinations (e.g., X-rays, pulmonary function diagnostics, CT)	Anxiety, stress [28]	Stress reduction, orientation	AKTIVER [22), The Elderly in the OR [91]

*CHOPs *Care of Confused hospitalised Older Persons, *CPB *Cardiopulmonary bypass, *CT *computed tomography, *ERAS *Enhanced Recovery After Surgery, *HELP *Hospital Elder Life Program, *help + *Hospital Elder Life Program Plus, *mHELP *modified Hospital Elder Life Program, *OR *operating room, *POD *Postoperative Delirium, *SBI *Safe Brain Initiative, *VR *virtual reality

#### Reduction of stressors/activation of resources

This module is about reducing stressors and activating the patient’s resources. Stress reduction is one of the core recommendations in the SBI [42]. This should be achieved, for example, by applying patients’ favorite music or relaxation music and by handing out noise-canceling headphones to decrease exposure to the many unfamiliar noises of a hospital environment. In addition, important biographical details, preferences, and resources are using the “Life Flower” tool, allowing for personalized patient support during the hospital stay. The Life Flower is an adaptation of the Sunflower Tool from the Care of Confused Hospitalised Older Persons (CHOPS) concept and is intended to ensure person-centered care [86, 92].

#### PLUS and ACTIVE visit

The PLUS and ACTIVE visits are focused on reorientation and information as well as cognitive activation. Older adults, particularly those with preexisting cognitive impairments, often experience challenges when adapting to new environments. Changes in the environment, along with factors such as surgery or illness itself, can lead to disorientation or disturbance of memory/attention [86]. The PLUS visit aims to prevent this by having the AKTIVER Heart team reorient patients regarding time, place, situation, and their person on a daily basis. Reorientation is a key component of many delirium prevention programs [22, 86, 89, 90] and reorientation interventions have been shown to reduce the risk of delirium [21]. To help maintain patients’ sense of orientation, the AKTIVER Heart team also ensures that essential aids, such as glasses, hearing aids, and dentures, are used. The main features of the ACTIVE module are comprised of cognitive activation tasks that last no longer than 20 min and give patients a sense of achievement. Examples of possible cognitive activation tasks include board games, crossword puzzles, general knowledge quizzes, reading stories aloud, listening to music, or looking at pictures [89]. A virtual reality headset (CUREO®, CUREosity GmbH, Duesseldorf, Germany) also can be used with various exercises for movement, directing attention, fine motor skills, and relaxation to keep patients occupied.

#### FIT visit

Promoting early mobilization after a surgical procedure is important in order to maintain the patients’ range of motion and reduce the risk of various complications (e.g., pneumonia, thrombosis, decubitus or constipation) [93]. As part of the FIT visit, patients will receive mobilization, exercises in bed, or breathing therapy by a physiotherapist. Unlike the AKTIVER and Help+ programs [22, 89], patients in the AKTIVER Heart group receive physical therapy twice a day instead of once a day during the first five postoperative days.

#### Meal accompaniment

An adequate supply of food and drink can also help to prevent POD [94]. Meal accompaniment is a key component in many delirium prevention programs [22, 86, 87, 89, 90]. The AKTIVER Heart team will support patients with their food intake depending on their needs to avoid malnutrition and stimulate appetite. Support is offered in the form of company at mealtimes, assistance with meal preparation, or general encouragement to eat and drink [22].

#### Promotion of sleep/relaxation

Many patients experience sleep disturbances in the hospital, often due to the unfamiliar environment, noises, restlessness, and anxiety [95]. In older patients, nighttime sleep disturbances can lead to daytime drowsiness and excessive daytime sleeping, resulting in a reversal of the sleep–wake cycle. The AKTIVER Heart team will promote sleep with non-pharmacological interventions, which primarily include calming measures such as hand massages, encouragement of sleep rituals, relaxation music, guided imagery, and the use of sleep masks [88, 89].

#### Diagnostic chaperonage

Before undergoing cardiac surgery, patients first undergo a multitude of preoperative examinations. These include chest X-rays, ECGs, lung function tests, CT scans of the chest, echocardiograms, and Doppler ultrasound of the neck vessels. Chest X-ray, ECG, and echocardiogram are often repeated postoperatively. These examinations are commonly associated with long distances and sometimes longer waiting times to help alleviate fears and provide guidance, as it is done in the delirium prevention program “The Elderly in the OR” [91].

#### TAU+ group

Before cardiac surgery, patients allocated to the TAU+ group (*n* = 20) will be accompanied by the study personnel to the preoperative examinations described above. Following randomization, they will receive a brief standardized preoperative education session covering the surgical procedure, the expected postoperative course, common postoperative symptoms, and the use of medical devices and drains commonly required after cardiac surgery. Patients will also receive standardized information about POD, including typical symptoms and its temporary nature.

As perioperative education of this kind is not part of current routine care at the study center, the control condition was defined as TAU+. Participants in both study groups receive identical standardized education on perioperative and postoperative procedures as well as POD symptoms. Patients in the AKTIVER Heart group additionally receive the multimodal intervention components, including preoperative exercises and breathing therapy, strategies to reduce distress and anxiety, and postoperative intervention elements.

The decision to provide standardized perioperative education to both study groups was made for both methodological and ethical reasons. Although educational interventions may influence patient knowledge, anxiety, treatment expectations, engagement in care, and potentially postoperative outcomes, including POD risk, standardized education is consistent with patient-centered care principles and current recommendations emphasizing informed decision-making and active patient participation in perioperative care. Furthermore, because perioperative education is not routinely delivered in a standardized manner at our institution, providing identical educational content to all participants reduces variability in usual care and enhances comparability between groups. Consequently, TAU+ serves as an active comparator, allowing the feasibility, acceptability, and potential added value of the multimodal AKTIVER Heart intervention to be evaluated beyond the effects of standardized education alone.

#### Study outcomes

The primary endpoints of the study are the feasibility and acceptability of the AKTIVER Heart intervention, as well as the feasibility of the study design (see Statistical Analysis Plan in Supplementary Material 5). Feasibility of the AKTIVER Heart intervention will be evaluated based on intervention delivery and adherence to predefined intervention components. Specifically, the intervention will be considered feasible if at least 90% of patients in the AKTIVER Heart group (*N* = 36) receive all of the following components: (i) preoperative information on perioperative procedures, POD, and preoperative somatic optimization by the cardiac surgeons; (ii) psychological preparation approximately 10–14 days before the planned procedure; and (iii) at least one intervention module per day after extubation for five postoperative days. In addition to the predefined composite feasibility outcome, we will report adherence to the individual intervention components, including completion rates for preoperative education, psychological preparation, and postoperative intervention modules. Acceptability focuses on measuring patient satisfaction with the AKTIVER Heart intervention and the involved clinical staff using five items rated on a numerical scale from 0 (not acceptable/useless) to 10 (highly acceptable/very helpful). The main acceptability statement is: “I was able to accept the therapeutic measures of the AKTIVER Heart delirium prevention program well during my inpatient stay.” Feasibility of the study design will be determined by the average number of patients randomized per week and the proportion of participants completing follow-up assessments. Secondary outcomes are exploratory, aiming to assess preliminary estimates compared to treatment as usual and to inform outcome selection and parameter estimates for a future trial. These clinical and patient-reported outcomes include POD incidence, severity and duration; intra- and postoperative complications; time-to-extubation; ICU and overall length of stay; 30- and 90-day mortality. Furthermore, measures of cognition (MoCA, 6-CIT), health-related QoL, frailty (CHSA CFS, handgrip strength), nutritional status (MNA), anxiety and depression (HADS), posttraumatic stress symptoms (IES-R), sleep quality (PSQI) and treatment expectations (IPQ, TEX-Q) are assessed.

#### Sample size

To monitor the feasibility of the AKTIVER Heart intervention, the proportion of patients completing at least one module per day over the course of 5 days (5-day completion rate) will be assessed. As a minimal requirement, we aim to show superiority over 50% completion rate [85, 96]; therefore, a one-sided binomial model will be applied at a significance level of 2.5%: H_0_: *p* ≤ 0.5 vs. H_1_: *p* > 0.5. Assuming a conservative 5-day completion rate of 75% and a sample size of 40 patients, the power of the underlying test is 90%. The completion rate can be estimated with an accuracy of ±13.6% (95% confidence interval [CI]). Calculations were performed using nQuery Version 9.2.1.0. To assess the feasibility of the study design, we assess the completion rate of the 3-month follow-up assessments. Assuming a true completion rate of 75%, the proportion of patients completing all 3-month follow-up assessments (*N* = 60, 2:1 allocation ratio) can be estimated with a precision of ±11% (95% CI). The acceptability of the AKTIVER Heart intervention is measured on a scale of 0 (not acceptable at all) to 10 (highly acceptable). Assuming a standard deviation of 3, the mean can be estimated with an accuracy of ±0.93 (95% CI width is 1.86).

### Statistical analysis

All outcomes will be evaluated following the intention-to-treat (ITT) principle using R (v. 4.3.1), with methodologies detailed in the pre-specified Statistical Analysis Plan (Supplementary Material 5). Feasibility of the intervention is considered achieved if at least 90% of patients in the AKTIVER Heart intervention group receive all three predefined core components of the intervention: (i) preoperative education on the perioperative process, POD, and preoperative somatic optimization, (ii) psychological preparation, and (iii) at least one AKTIVER Heart module per day after extubation for five postoperative days. Acceptability will be achieved if the mean patient rating on a 5-item satisfaction scale (0 = not acceptable, 10 = highly acceptable) is ≥7.0, supplemented by qualitative interviews for future program adaptation. Feasibility of the study design requires randomizing an average of at least one patient per week over 36 weeks and retaining ≥75% of participants through the 3-month follow-up. For secondary endpoints, descriptive statistics and effect estimates with 95% confidence intervals (CIs) will be prioritized. Dichotomous outcomes will be analyzed using logistic regression (reporting odds ratios), count outcomes via Poisson or negative binomial regression (rate ratios), and time-to-event outcomes using Cox regression (hazard ratios). Continuous endpoints will be analyzed using generalized linear mixed models (GLMMs) to calculate estimated marginal means adjusted for baseline values. Because analyses are exploratory, any reported *p*-values are solely descriptive and will not serve as confirmatory evidence. Additional subgroup analyses (e.g., by age or sex) will be conducted as part of associated doctoral theses.

### Data management

Data entry will be conducted via the REDCap database, an established Good Clinical Practice-compliant web solution [97]. REDCap is specifically configured for the PREVENT-POD study, allowing secure and pseudonymous data storage. Checks for data plausibility and missing data are performed throughout the study to ensure high data quality and monitor recruitment. After the publication of the primary results, access to the anonymized data for legitimate research purposes will be provided by a Data Access Committee to fulfill the Findability, Accessibility, Interoperability and Reuse (FAIR) principles [98].

## Discussion

In older patients undergoing cardiac surgery, POD is a common complication with an incidence rate of up to 55% [4, 12, 18, 99]. POD is often associated with prolonged stay in the ICU and hospital, higher rates of institutionalization, postoperative complications such as pneumonia and falls, increased risk of cognitive impairments and dementia, and higher mortality [11, 14, 18]. Compared to other surgical fields, patients undergoing cardiac surgery often face additional factors that increase the risk of POD [26], including long surgery duration, intraoperative hypoxemia, the need for blood transfusions, duration of inotropic medication administration, longer ventilation time, use of cardiopulmonary bypass, prolonged ischemic time, more complex surgical procedures, or atrial fibrillation [5, 7, 8, 12, 26, 99]. These must be considered when preventing POD following cardiac surgery.

Multimodal, non-pharmacological delirium prevention interventions can significantly reduce delirium incidence by up to 40% [21, 22, 24]. In the multicenter, stepped-wedge cluster randomized PAWEL study [25], the AKTIVER intervention resulted in significant reductions in both POD incidence and the number of POD days in patients undergoing orthopedic and visceral surgery [22]. However, it did not significantly impact post-surgical outcomes in individuals undergoing cardiac surgery. Accordingly, we adapted the AKTIVER intervention to cardiac surgery populations by adding a perioperative optimization module. This module was informed by the ERAS program [81], which optimizes the preparation and recovery phases of surgery [81, 99]. ERAS has been associated with reduced health costs [100], shorter hospital LOS, shorter ventilation time, and reduced rates of postoperative opioid prescriptions [101]. Given these achievements, we anticipate that the ERAS-inspired perioperative optimization modules, in combination with the existing modules of the AKTIVER intervention, have the potential to reduce the incidence and severity of POD also in cardiac surgery population. The PREVENT-POD pilot study is a randomized controlled trial that evaluates the feasibility and acceptability of the AKTIVER Heart intervention in elderly patients (≥65 years) undergoing elective cardiac surgery. With a sample size of 60 participants, divided into AKTIVER Heart (*n* = 40) and TAU + groups (*n* = 20), data is collected over a period of more than 3 months. The primary objectives are to assess the feasibility and acceptability of the AKTIVER Heart intervention as well as the feasibility of the study design. Additionally, preliminary estimates will be obtained concerning clinical outcomes such as POD incidence, cognitive functions, and QoL. In addition to evaluating feasibility, acceptability and preliminary estimates, the PREVENT-POD pilot trial will generate important implementation data regarding intervention delivery, resource utilization, staffing requirements, workflow integration, and potential implementation barriers. These data will directly inform the design, staffing model, training procedures, and implementation strategy of a future multicenter RCT.

The PREVENT-POD study has several key strengths. First, by assessing the feasibility of implementing the interventions in a small patient sample. Second, by integrating perioperative optimization into the AKTIVER multimodal POD-prevention framework, the AKTIVER Heart interventions addresses individual risk as well as both general and procedure-specific POD risk factors in a single pathway. Third, delivery by a specialized and trained interprofessional team—surgeons, nurses, a psychosomatic expert, physical therapists, and pharmacists–will ensure high-fidelity implementation and facilitate strong patient engagement. Finally, the inclusion of both qualitative interviews and quantitative measures across POD, cognition, mood, sleep, and function will generate comprehensive insights to refine the intervention prior to efficacy testing in a future multicenter trial. Potential limitations of the present pilot study include the intensity and complexity of the multimodal intervention, which may pose challenges for future implementation in routine clinical care. In addition, the relatively small sample size is appropriate for a feasibility pilot trial but limits the statistical power to detect effects on clinical efficacy outcomes. Furthermore, the baseline assessment window ranging from 2 days to 3 weeks before surgery may introduce variability in patients’ preoperative psychological state. In particular, anxiety may increase as the date of surgery approaches and could adversely affect concentration and performance on cognitive assessments such as the MoCA, as well as self-reported outcomes. Consequently, baseline measures may partly reflect differences in assessment timing rather than true interindividual differences. This potential source of measurement variability should therefore be considered when interpreting baseline findings and exploratory outcome analyses. Another limitation is that blinding of outcome assessors after baseline was not feasible within the pragmatic ward-based study setting. As the same trained personnel conducted follow-up assessments and delivered the intervention during their shifts, knowledge of treatment allocation may have introduced detection bias, particularly for outcomes requiring clinical judgment or observer ratings, such as delirium assessments. Although standardized and validated delirium instruments are used, observer bias and expectancy effects cannot be fully excluded, particularly for delirium outcomes assessed during the postoperative period. Consequently, the exploratory efficacy findings should be interpreted with caution. Future adequately powered RCTs should incorporate more rigorous blinding procedures, including independent outcome assessors and/or video-recorded assessments scored by blinded raters, to reduce the risk of detection bias and strengthen the validity of delirium outcome assessments. Ultimately, a larger multicenter randomized controlled trial will be required to determine the efficacy of the AKTIVER Heart intervention and its impact on psychological, functional, and health-related outcomes in this high-risk population.

### Trial status

Recruitment of participants began on November 1, 2024. The first intervention period started on November 15, 2024. The last intervention was performed in October 2025.

### Dissemination

Upon completion of data collection, the data will be analyzed and published as part of several medical doctoral theses. The main findings will be published in peer-reviewed journals and presented at conferences.

## Supplementary Information

Supplementary Material 1. AKTIVER Heart intervention manual.

Supplementary Material 2. Instructions for breathing and movement exercises.

Supplementary Material 3. SPIRIT checklist.

Supplementary Material 4. Ethical Approval.

Supplementary Material 5. Statistical Analysis Plan.

## Data Availability

The PREVENT-POD dataset will be available upon request from the corresponding author (MS) and the study statisticians (CD, TA). The study investigators follow the FAIR principles for scientific data management and stewardship [98].

## Abbreviations

ACB: Anticholinergic Burden
ACC: Aortic Cross Clamping
ADL: Activities of daily living
AKTIVER: Alltags- und Kognitionstraining und Interdisziplinarität Verbessert das Ergebnis und mindert das Risiko
AMT-4: Abbreviated Mental Test-4
CAM: Confusion Assessment Method
CFS: Clinical Frailty Scale
CHOPS: Care of Confused Hospitalised Older Persons Program
CI: Confidence Interval
CPB: Cardiopulmonary Bypass
DSM-V: Diagnostic statistical manual fifth version
DRS-R-98: Delirium Rating Scale-Revised-98
DRSQ: Delirium Risk Screening Questionnaire
EQ-5D-5L: Five-dimensional Europan Quality of life Questionnaire
ERAS: Enhanced Recovery After Surgery
FAIR: Findability, Accessibility, Interoperability and Reuse principles
FINDERI: FIND Delirium Risk Factors
FU: Follow-Up
HeartQoL: Heart Quality of Life
HELP: Hospital Elder Life Program
HADS: Hospital Anxiety and Depression Scale
I-CAM-S: ICD-10-Confusion Assessment Method-Severity
ICU: Intensive care unit
IPQ-B: Illness Perception Questionnaire
LOS: Length of Stay
MNA-SF: Mini Nutritional Assessment—Short Form
MoCA: Montreal Cognitive Assessment
OR: Odds Ratio
PSQI: Pittsburgh Sleep Quality Index
POD: Postoperative delirium
PREVENT POD: Prevent postoperative Delirium
RR: Risk Ratio
SARC-F: Strength, Assistance with walking, Rise from a chair, Climb stairs and Falls
SBI: Safe Brain Initiative
SMI: Subjective memory impairment
TAU +: Treatment as usual + information about perioperative procedures and delirium
TEX-Q: Treatment Expectation Questionnaire
UMG: University Medical Center Goettingen
4-AT: 4 A’s Test
6-CIT: 6-Item Cognitive Impairment Test
