# Supplementary material for: Feasibility, acceptability, and preliminary estimates of a multimodal AKTIVER Heart Delirium Prevention Program in patients indicated for cardiac surgery: study protocol of a randomized controlled PREVENT POstoperative Delirium (PREVENT-POD) Pilot Study
Source: Pilot Feasibility Stud. 2026 Oct 5;12:142. doi: 10.1186/s40814-026-01923-6 (PMC13637325; doi:10.1186/s40814-026-01923-6)
Supplement: Supplementary file 1 [file 40814_2026_1923_MOESM1_ESM.pdf]

Treatment and Training Manual

PD Dr. med. Christine Thomas

Juliane Spank, M.A.

PD Dr. med. Monika Sadlonova

Prof. Dr. med. Christine von Arnim

Prof. Dr. med. Ingo Kutschka

Prof. Dr. med. Hassina Baraki

Dr. Sportwiss. Carolin Steinmetz

Stuttgart and Göttingen

Version 1.0 from 08/28/2024

Klinikum Stuttgart

UNIVERSITÄTSMEDIZIN GÖTTINGEN UMG

Table of content

1. AKTIVER Heart delirium prevention program 1
    1.1 Expectations placed on the AKTIVER Heart intervention team 2
2. Modules of the AKTIVER Heart delirium prevention program 3
    2.1 Preoperative (somatic) preparation 3
        2.1.1 Information about the perioperative procedure and delirium 3
        2.1.2 Respiratory therapy 5
        2.1.3 Sports/movement therapy 6
        2.1.4 Perioperative optimization 8
    2.2 Preoperative (psychological) preparation 11
        2.2.1 Psychological preparation with a focus on anxiety reduction and education 11
        2.2.2 Reduction of stressors and activation of resources 13
    2.3 PLUS Visit and Active Visit 14
    2.4 FIT Visit 16
    2.5 Assistance with meals 18
    2.6 Relaxation and sleep promotion 20
    2.7 Accompaniment to diagnostic appointments 23
3. Cognitive, psychological and behavioral changes 24
    3.1 Challenging behavior 24
4. Communication and conversation 26
    4.1 Multi-layered communication 26
    4.2 Verbal and non-verbal communication 27
    4.3 Communication in cases of delirium or cognitive dysfunction 27
    4.4 Validation and Integrative Validation according to Richard® 28

1. AKTIVER Heart delirium prevention program

The "AKTIVER Heart" program was developed as part of the HuBerTDA and PAWEL projects at Stuttgart Hospital and the PRECOVERY, I-COPE, and FINDERI studies at the University Medical Center Göttingen. It aims to improve the hospital experience of elderly patients undergoing cardiac surgery and prevent the occurrence of postoperative delirium.

A hospital stay is usually a major event for everyone and can, depending on the circumstances, have undesirable effects. For people aged 65 and over in particular, these often lead to a deterioration in physical and mental abilities, which can make a full recovery more difficult.

Hospital stays for older people often lead to a loss of their previous level of function in everyday life. For some patients, discharge from the hospital means they are no longer able to live at home and are subsequently admitted to a care home. Some of these unwanted deteriorations can be effectively prevented. The "AKTIVER Heart" delirium prevention program is designed to plan and ensure this.

The goals of this program are:

- To help patients maintain their mental and physical functions throughout their hospital stay in cardiac surgery.
- To prevent the occurrence of postoperative delirium.
- To reduce complications that cause further medical interventions.
- To enable patients to be as independent as before after discharge.

The AKTIVER Heart Intervention Team (PD Dr. Sadlonova, Dr. Steinmetz, Ms. Kühnle, Mr. Dong, Mr. Heißenberg, Ms. Badde, Ms. Schettler, Ms. Neitzel, Ms. Viehmeister, Prof. Baraki, Mr. Botnariuc, Dr. Itting) facilitates interactions with patients that would otherwise not occur. The AKTIVER Heart Intervention Team provides therapy by offering support and companionship to elderly patients before and after heart surgery. Through seven specific interventions, they assist older people with an indication for planned cardiac surgery:

1. Preoperative (somatic) preparation
2. Preoperative (psychological) preparation
3. ACTIVE visit and PLUS visit
4. FIT visit

5. Assistance with meals
6. Relaxation and sleep promotion
7. Accompaniment to the examinations

The individual interventions are presented in Chapter 2

1.1 Expectations placed on the AKTIVER Heart intervention team

To achieve the goals of the "AKTIVER Heart" program, the study team should meet high-performance standards and put them into practice:

- Reliability

Patients must be able to rely on interventions being carried out reliably throughout their hospital stay. Punctual and reliable attendance during scheduled working hours is expected as a matter of course. In the event of illness or other important reasons for absence, the other members of the study team should be informed as early as possible so that a replacement can be planned.

- Respecting your boundaries

The "AKTIVER Heart" intervention team must know and respect the limits of their role. Medical information or advice may only be given by the medical staff in the study team (PD Dr. Sadlonova, Prof. Baraki, Mr. Botnariuc, Dr. Itting, PD Dr. El-Essawi). If a patient asks questions about their disease, therapy, or similar, these must be passed on to the treating physicians or the medical staff of the PREVENT-POD study. The AKTIVER Heart study team is only permitted to contribute to the medical treatment within a defined framework (perioperative optimization) in close cooperation with the attending physicians at the Clinic for Cardiothoracic and Vascular Surgery at the University Medical Center Göttingen. Any restrictions regarding food and fluid intake or mobilization must always be observed and clarified with the specialist in case of uncertainties.

- Good judgment

Working independently with patients requires a high degree of reflection and solution-oriented action. If it is unclear how to act in a situation, you must consult the study director or deputy study director in advance.

- Confidentiality

The handling of patients' medical and personal information is subject to a duty of confidentiality towards everyone who is not directly involved in the treatment. This must also be observed when passing on information to relatives.

- Respect

Encounters with patients and relatives must be guided by appreciation and respect. The patient must always be informed in advance of any activities and must consent to the measure. Greet patients appropriately with their surname.

- Personal appearance

The AKTIVER Heart Intervention Team, like all employees, must maintain a clean, professional appearance. The name badge must always be worn legibly, and hospital clothing is mandatory. The guidelines for hospital staff apply regarding jewelry, make-up, etc.

2. Modules of the AKTIVER Heart delirium prevention program

2.1 Preoperative (somatic) preparation

This module aims to better prepare patients for their planned cardiac surgery. This involves not only providing information about the procedure and postoperative delirium. Patients are also physically prepared for the surgical procedure with the help of physiotherapy and respiratory therapy. In addition, the practitioners at the Clinic for Cardiothoracic and Vascular Surgery will pay particular attention to optimizing pre-, intra-, and postoperative, potentially delirogenic factors to minimize them accordingly.

2.1.1 Information about the perioperative procedure and delirium

Implementation: Physicians of the AKTIVER Heart team

Objectives: Education about

- The present cardiac disease
- The procedure for admission to hospital (pre-operative examinations)
- The planned cardiac surgery
- The postoperative process in the intensive care unit and normal ward
- Follow-up treatment
- Symptoms of postoperative delirium and the role of cognitive training

Further information about cardiac surgery:

- Patients are usually initially treated in the intensive care unit for 1-2 days and extubated a few hours after the procedure. Important aspects are noise, partial restriction of privacy, the feeling of being dependent on others, feelings of shame, anxiety (e.g. also when fellow patients are suffering), and sleep disorders.
- Drains: As a rule, 2-3 drains are placed. Patients should breathe in well and hold their breath during removal.
- Indwelling catheter: Is left in place for approx. 2-3 days (shorter if possible)
- Temporary pacemaker: precautionary measure during cardiac surgery. This is a thin cable that is usually removed on the 4th postoperative day or earlier. Here too, patients should breathe in well and hold their breath during removal.
- Postoperative pain: Especially during the first 3-4 days, patients should ask for medication if they are in pain.
- If coughing/expectoration occurs: The chest can be stabilized with a rolled towel and with arms crossed in front of the chest. This gives a feeling of security.
- After cardiac surgery, monitoring (so-called "telemetry") is required to rule out cardiac arrhythmia (e.g. atrial fibrillation).
- Occurrence of postoperative delirium: In some cases, up to 50% of patients undergoing heart surgery experience symptoms such as altered perception, cognitive impairment, confusion, agitation, a shift in the day-night rhythm, adynamia, or endangerment of themselves or others.

Further information:

- Early mobilization and breathing training (already in the intensive care unit).
- Initial administration of a high number of medications (especially in the first few days - painkillers, magnesium, potassium, etc.), which will be reduced gradually.
- Importance of good communication with the treating doctors and healthcare professionals ("It is important to be informed and educated about the state of health").
- After the procedure, follow-up cardiac rehabilitation is recommended.
- Information about the symptoms of postoperative delirium that can occur after cardiac surgery.

Precautionary measure: Specific questions from patients are addressed. Furthermore safety, trust, and professionalism are conveyed.

2.1.2 Respiratory therapy

Implementation: Physiotherapists and sports scientists and, after initial instruction, Patients themselves

Objectives: Development of breathing awareness, enlargement, and perception of breathing spaces, economization of breathing technique, learning various breathing techniques, strengthening of the inspiratory respiratory muscles to reduce postoperative pulmonary complications, and instruction in self-training.

Execution: All patients receive the "Coach 2" breathing trainer preoperatively. They are asked to complete 10 breathing cycles 6 times a day. The aim is to inhale via the mouthpiece so that the yellow sheet is held in the marked area of the smiling face for as long as possible. This is followed by a slow exhalation via the lip brake. In the lip brake, the lips are pursed slightly. The resulting resistance prolongs the exhalation. Patients should take breaks of up to two minutes between breathing cycles.

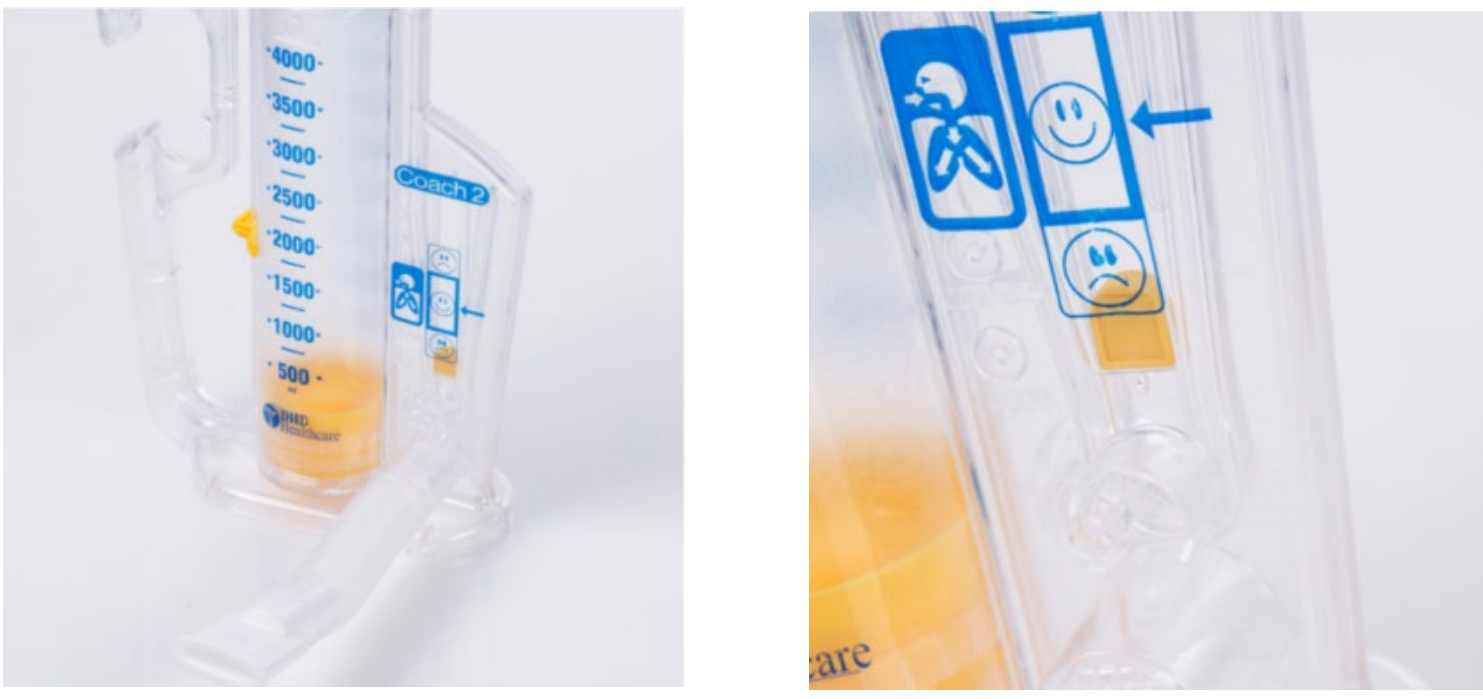

Figure 1 COACH 2 Breathing trainer https://www.lungentrainer.de/atemtrainer-coach-2.html

In addition, patients are provided with an interactive video and a brochure containing further breathing exercises. The instructions in the video are given by a qualified specialist (physiotherapist).

The main components of preoperative respiratory therapy in addition to self-training with "Coach 2" are:

- ✓ Learning to breathe deeply and consciously (chest, abdominal, flank, and back breathing)
- ✓ Learning various breathing techniques, such as the lip brake

We recommend a maximum of 6-8 breathing cycles per exercise in combination with subsequent active breaks (see video, brochure). We recommend doing the exercises in the video 3-5 times a week.

Precautionary measure: It is important to do the exercises in such a way that no symptoms such as dizziness or discomfort manifest.

2.1.3 Sports/movement therapy

Aerobic endurance training

Implementation: After initial instruction by physiotherapists or sports scientists, the self-training takes place.

Objectives: Stabilization/increase in cardiopulmonary performance, reduction of cardiopulmonary complaints, strengthening of body awareness, improvement in perioperative quality of life, reduction in postoperative immobilization, optimization of cardiovascular risk factors.

Execution: It is recommended to do moderate aerobic endurance training at least 3 times a week for 10-60 minutes - depending on the patient's fitness level - in the form of walks on level ground. The intensity should allow continuous activity without undue strain. The subjective feeling of exertion can also be used as a control parameter. You should aim for a range between 11-14, easy to somewhat strenuous.

Score	Rating of Perceived Exertion
6	
7	Very, very light
8	
9	Very light
10	
11	Fairly light
12	
13	Somewhat hard
14	
15	Hard
16	
17	Very hard
18	
19	Very, very hard
20	

Figure 2 15-level-Borg Scale

Make sure that the training load during aerobic endurance training is within the symptom free range.

Contraindications for aerobic endurance training:

- ! Malignant hypertension with blood pressure increases to systolic >190 mmHg during exercise load despite maximum possible antihypertensive combination therapy.
- ! Drop in blood pressure of systolic ≥20 mmHg under stress, especially in patients with CHD.
- ! Hemodynamically compromising supraventricular and ventricular arrhythmias, persistent ventricular tachycardia.
- ! Frequent ventricular extrasystole, non-sustained ventricular tachycardia in advanced left ventricular dysfunction and after myocardial infarction as well as after physical exertion or in the post-exercise phase.

Mobilization and strengthening

Implementation: After initial instruction by physiotherapists or sports scientists, the self-training takes place.

Objectives: Improving strength, increasing intermuscular coordination (fall prevention), training body awareness, optimizing muscle metabolism, reducing post-operative immobilization, improving mobility, increasing economy of movement, and facilitating everyday activities.

Execution: Mobilization and strength training with the aid of a chair/stool. The exercises are modified according to the evidence-based OTAGO fall prevention program. The focus is on strengthening the lower extremities. The program is supplemented by mobilization exercises for the upper extremities. It is recommended to complete the training at least 3 times a week. The training can be completed with the help of the video or the brochure.

Precautionary measure: Make sure that the training load during the exercises is within the symptom-free and pain-free range. Interrupt the training if pain/discomfort of any kind occurs. It is also important to ensure that all exercises are carried out in a normal breathing rhythm (without forced breathing).

The above-mentioned precautions for aerobic endurance training also apply, as well as the following contraindications for performing strengthening exercises:

- ! Difficult to adjust hypertension e.g. permanently above 160/100 mmHg
- ! Severe pulmonary hypertension e.g. pulmonary artery pressure of over 50 mmHg
- ! Critical thoracic / abdominal aortic aneurysm
- ! Early-stage ventricular aneurysm

2.1.4 Perioperative optimization

Implementation: Attending physicians in the Clinic for Cardiac, Thoracic and Vascular Surgery

Objectives: Optimization of intra- and postoperative procedures with a special focus on reducing potential delirogenic factors.

Execution: Attending physicians in the AKTIVER Heart intervention group will closely monitor compliance with the perioperative aspects (part of the measures is shown in **Figure 3** and **Figure 4**). In the pre- and perioperative procedures, the attending physicians at the Clinic for Cardiothoracic and Vascular Surgery will focus on optimizing the iron balance (incl. anemia), blood pressure, hypoalbuminemia (nutritional status), and other parameters if there are indications of malnutrition (vitamin B12, vitamin D, BMI), preoperative risk assessment (*American Society of Anesthesiologists,* and EUROScore II) and the reduction of anticholinergic burden (*anticholinergic burden score*, ACB score < 3, **Table 1**).

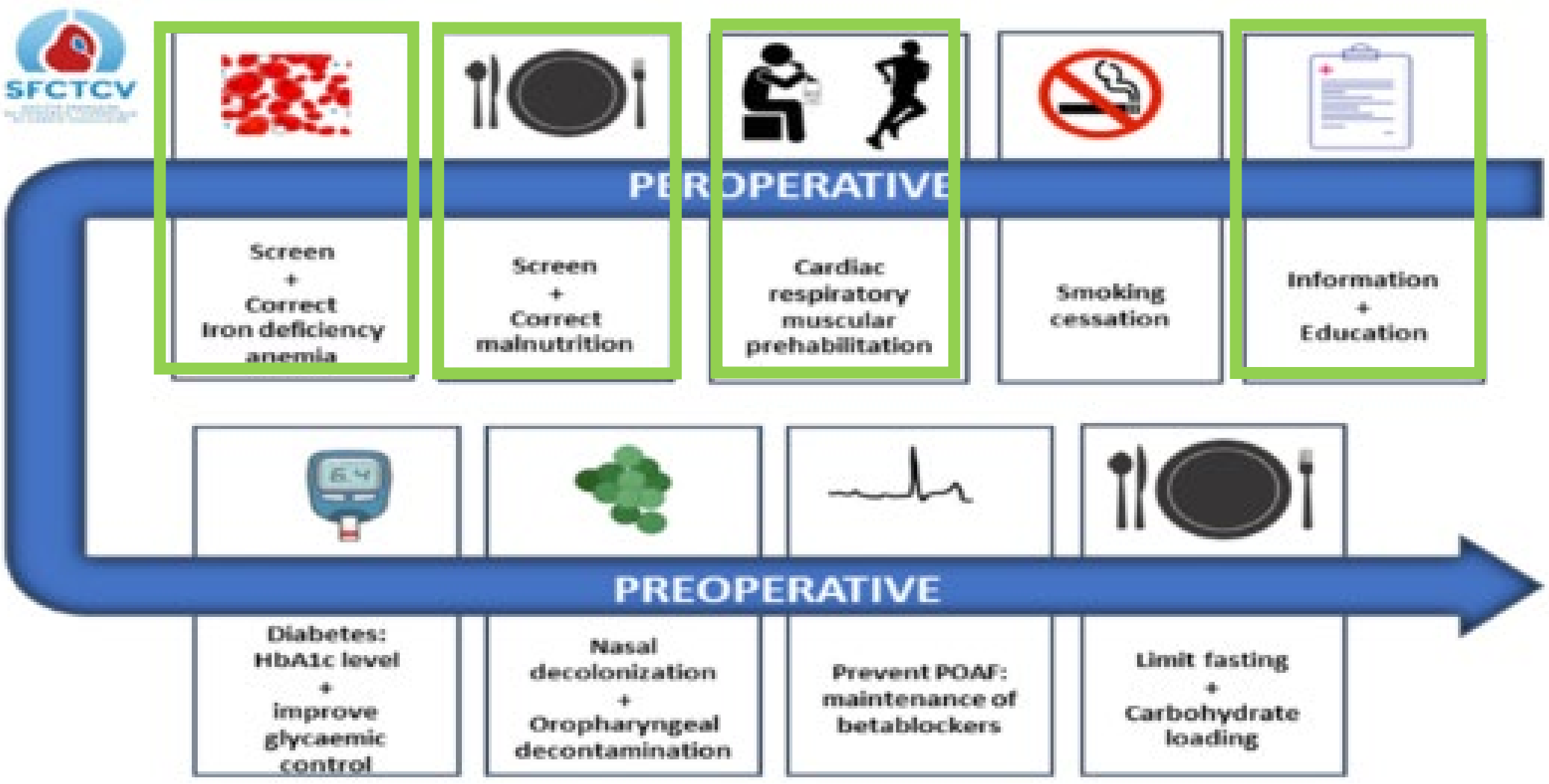

Figure 3 Preoperative (somatic) optimization measures are marked in green (mod. After Mertens et al. 2022; Grant et al. 2024; Engelmann et al. 2019)

Intraoperatively, the attending physicians of the AKTIVER Heart Intervention Team will also pay attention to reducing potentially delirogenic intraoperative factors and repeatedly draw the attention of the treatment team to this. Particular attention is paid to the duration of cardiac surgery, optimization of volume management, reduction of hypotensive phases (neuromonitoring?), venting of the left ventricle when the heart-lung machine is removed, and ischemia time. These measures are already part of a recommended routine, e.g. as part of the *Enhance Recovery After Surgery* (ERAS) guidelines (Grant et al. 2024) and are described in several publications as potentially delirogenic intraoperative aspects.

In addition, AKTIVER Heart teams will ask the attending physicians to optimize postoperative procedures (part of the strategies is shown in Figure 3): *early extubation* (if possible extubation within 6-8 hours after surgery), reduction of anticholinergic load - **ACB score < 3**, reduction of opiates and benzodiazepines for sedation, if possible no administration of benzodiazepines to promote sleep, earliest possible postoperative fluid and food intake and administration of regular medication, administration of long-term medication - especially no discontinuation of antidepressants (CAVE: Venlafaxine and SSRI with discontinuation phenomenon), opiates and benzodiazepines (CAVE: withdrawal symptoms when discontinuing after surgery), removal of urinary catheters and surgical drains as early as possible and reduction of opiates in pain medication.

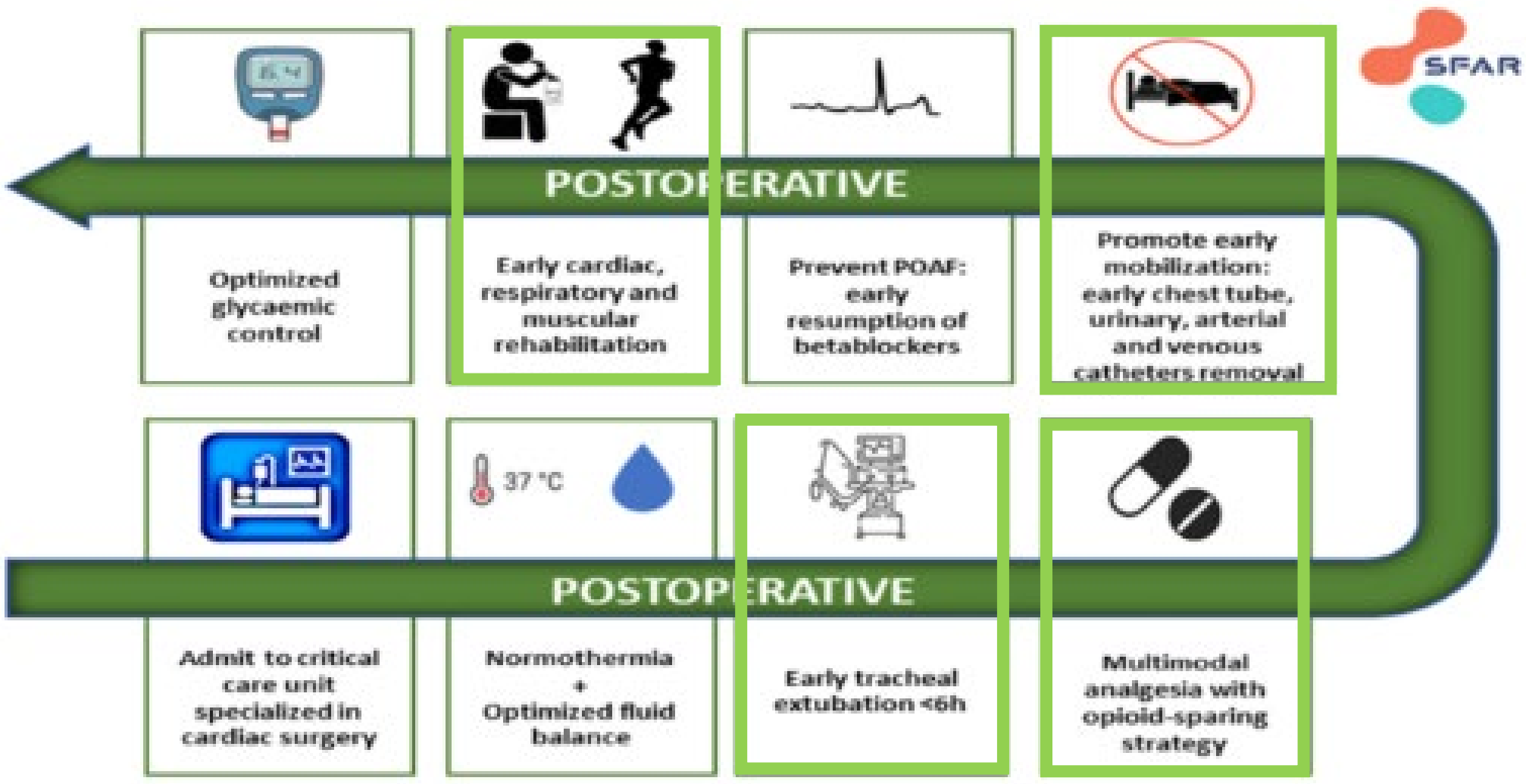

Figure 4 Postoperative (somatic) optimization measures are marked in green (mod. After Mertens et al. 2022; Grant et al. 2024; Engelmann et al. 2019

The above-mentioned pre-, intra-, and perioperative aspects are documented by the physicians to objectify compliance with the recommended procedures. Through the intensified follow-up of perioperative measures by the physicians of the AKTIVER Heart team from the Department of Cardiac, Thoracic and Vascular Surgery in patients aged 65 and older, we hope to achieve a reduction in the risk and incidence of delirium. Precautionary measure:

All measures may only be carried out after consultation with the responsible surgeon or senior physician in the intensive care unit:

- ! Swallowing disorders
- ! Insufficient sedation
- ! Agitation after extubation
- ! Pain

Table 1 Anticholinergic Burden (ACB) score for drugs approved in Germany.

ACB score = 1			ACB score = 2	ACB score = 3
Aclidinium[inh]	Entacapone	Mirtazapine	Amantadine	Amitriptyline
Alprazolam	Escitalopram	Morphine	Carbamazepine	Atropine
Ampicillin	Etoricoxib	Naratriptan	Cimetidine	Chlorpheniramine
Aripiprazole	Famotidine	Nifedipine	Haloperidol	Clemastine
Asenapine	Fentanyl	Oxazepam	Loperamide	Clomipramine
Atenolol	Fexofenadine	Oxycodone	Loxapine	Clozapine
Azathioprine	Flunitrazepam	Paliperidone	Maprotiline	Cyproheptadine
Baclofen	Fluoxetine	Pancuronium	Methadone	Darifenacin
Benazepril	Fluphenazine	Perphenazine	Olanzapine	Dimenhydrinate
Betaxolol	Flurazepam	Phenobarbital	Opipramol	Diphenhydramine
Bisacodyl	Fluvoxamine	Piperacillin	Oxcarbazepine	Doxepin
Bromocriptine	Furosemide	Pramipexole	Paroxetine	Fesoterodine
Bupropion	Gentamicin	Prednisolone	Pethidine	Flavoxate
Captopril	Glycopyrronium[inh]	Prednisone	Pimozide	Hydroxyzine
Celecoxib	Guaifenesin	Promethazine	Quetiapine	Imipramine
Cetirizine	Hydralazine	Pseudoephedrine	Ranitidine	Levomepromazine
Chlordiazepoxide	Hydrocortisone	Quinidine	Theophylline	Nortriptyline
Chlorthalidone	Ipratropium[inh]	Risperidone	Tramadol	Orphenadrine
Ciclosporin	Isosorbide dinitrate	Rotigotine patch		Oxybutynin
Citalopram	Isosorbide mononitrate	Selegiline		Procyclidine
Clindamycin	Ketorolac	Sertraline		Propiverine
Clonazepam	Lansoprazole	Sumatriptan		Scopolamine
Clorazepate	Levocetirizine	Temazepam		Solifenacin
Codeine	Levodopa	Tiotropium[inh]		Thioridazine
Desloratadine	Lithium	Trandolapril		Tizanidine
Dexamethasone	Loratadine	Trazodone		Tolterodine
Dextromethorphan	Lorazepam	Triamcinolone		Trihexyphenidyl
Diazepam	Metformin	Triamterene		Trimipramine
Digitoxin	Methocarbamol	Triazolam		Trospium
Digoxin	Methotrexate	Valproic acid		
Diltiazem	Methylprednisolone	Vancomycin		
Dimetindene	Metoclopramide	Venlafaxine		
Dipyridamole	Metoprolol	Warfarin		
Domperidone	Midazolam	Ziprasidone		
Doxylamine		Zolmitriptan		

[inh] inhalative

2.2 Preoperative (psychological) preparation

2.2.1 Psychological preparation with a focus on anxiety reduction and education

Implementation: Psychosomatic medicine physicians of the AKTIVER Heart team.

Objectives: The therapeutic goals of psychological discussions prior to a planned surgical procedure can be categorized as follows: **Management of affective comorbidity** (e.g. reduction of anxiety and depression), **strengthening of self-management** (e.g. strengthening of therapy motivation, development of appropriate/positive health beliefs, acceptance of responsibility), **improvement of postoperative quality of life** (e.g. optimization of coping with illness with resource activation, gaining confidence in the functioning of one's own body). This intervention provides psychological preparation for the upcoming heart surgery. In addition, educational and application-oriented components are designed to provide patients with long-term support in the further course of their health and improve coping with illness.

Execution: **Assessment of psychosocial status** (social support, sleep quality, psychological comorbidities, distress due to physical complaints, cognitive limitations, psychopharmacological treatment); **reduction of anxiety** (fear of expectation, fear of death, and fear of complications by means of cognitive restructuring), **psychoeducation** (developing the patient's subjective disease model, explaining the purpose of stress management, psychotherapy and psychotropic drugs, emphasizing the importance of adherence, activating social support, developing realistic expectations regarding the postoperative phase and intensive care unit), delirium education.

Building treatment motivation (elements of motivational interviewing), systemic questions for a solution-oriented approach, correction of dysfunctional disease assumptions, strengthening self-efficacy, and consideration of the patient's autonomy limits and individual preferences. Furthermore, imagination exercises involving the patient's personal interests and wishes, developing coping strategies, highlighting the advantages of surgery compared to the initial situation, and emphasizing the regained control over the heart disease (behavior-related outcome expectation).

As part of preoperative psychological preparation, a psychological session with standardized content based on the treatment concept of the PSY-HEART study and the I-COPE study will take place.

Session (duration: 30-50 min): "Heart surgery - newfound freedom and lifesaver"

At the beginning of the session, the patient is informed about the procedure, the timeline, and the goals of the psychological interventions. Patients are then informed that their attitudes, mental state, and expectations play an important role in their recovery. The focus of the first session is on reducing treatment-related fears and fears about the future, the subjective model of illness, and the calming effect on patients in relation to the upcoming operation (e.g. focus on a calming tone of voice). A basic understanding of the disease, the associated symptoms, and the medical intervention is developed with the patient based on their subjective disease/treatment model.

To build up the highest possible and most positive treatment-related outcome expectations, it is advisable to convey to the patient that the procedure will improve the current stressful and restrictive circumstances of heart disease. It should be made clear that the upcoming intervention/surgery will reduce the undersupply of oxygen to the heart (e.g. bypass surgery) or the restriction of heart valve function, improve associated symptoms (e.g. tightness of the chest) and improve physical performance. By discussing the course of the cardiac procedure and the time afterward, structural and procedural expectations are addressed, and the associated fears are reduced. These represent an

important aspect of the treatment context. In addition, misconceptions and assumptions (e.g. "My heart is broken and will never function properly again") are corrected. The therapists emphasize that the surgery is frequently performed and therefore safe procedure. The competence of the surgeons and the entire hospital staff is also described positively (formulated in patient-oriented language, e.g. "They are professionals. They do it every day and have a lot of experience."). Positive outcome expectations are also promoted primarily through information about symptom improvement, an increase in quality of life, and a positive-realistic recovery process by also explicitly addressing expectations regarding the timeline.

2.2.2 Reduction of stressors and activation of resources

Implementation: Research assistants and treatment assistants of the AKTIVER Heart team.

Objectives: To activate personal resources and reduce stressors before cardiac surgery using aids such as noise-canceling headphones, music players, and VR-guided relaxation.

Execution: The "AKTIVER Heart" team will guide patients through the so-called "Flower of Life" to identify key personal resources and preferences (see **Figure 5**). In addition, the patient's preferred VR scenario is recorded (especially for relaxation and exercise). The "AKTIVER Heart" team will also provide patients with noise-canceling headphones and MP3 players and demonstrate their use. Patients are asked to bring the provided devices with them to the session.

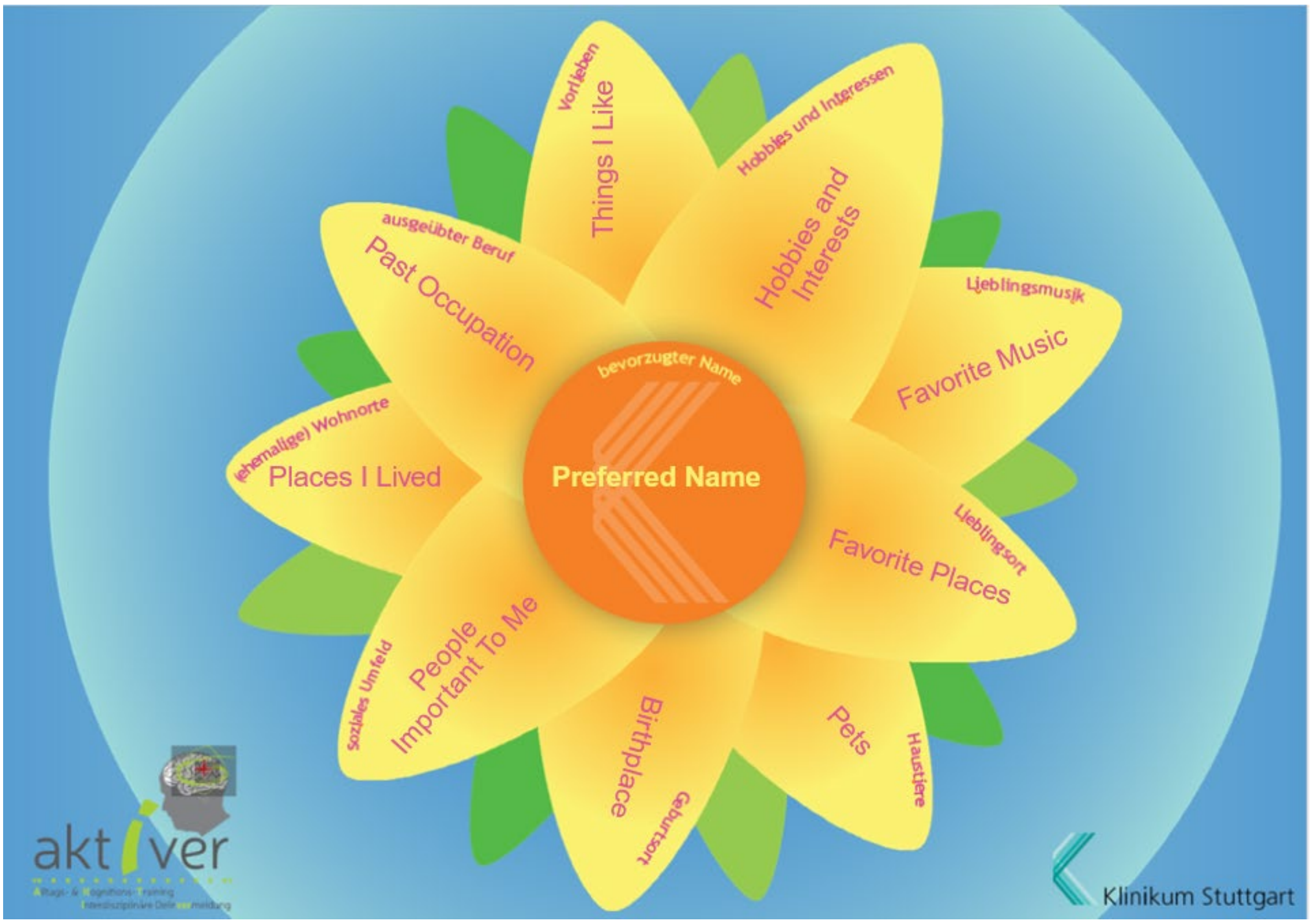

Figure 5 Flower of Life for surveying patient preferences and resources.

2.3 PLUS Visit and Active Visit

Implementation: Research assistants and treatment assistants.

Objectives: Older hospitalized people often become disoriented and forgetful even if they show no signs of confusion at home. If deficits already exist, these can worsen considerably. There are many reasons for this; the illness itself, treatment measures (surgery, examinations, infusions, etc.), new medication, but also unfamiliar surroundings, changes in daily routines, and many new people to adjust to. The "Active Visit" module aims to reduce confusion and promote well-being through reorienting older patients.

Execution: The “AKTIVER Heart” team will reorient patients daily after cardiac surgery. Patients are encouraged to use their own watch. The orientation covers four aspects:

- Time (Date, day of the week, season)
- Location (Where am I?)
- Situation (What am I doing here?)
- Person (Who am I?)

To give older patients sufficient orientation, important information is communicated, and guidance is offered. This is done in a planned, systematic, and continuous manner. Daily visits and dialogue provide patients with important information, for example, upcoming examinations.

Patients are regularly offered a drink and assisted with glasses, hearing aids, or dentures as needed. In the hospital, patients often suffer from boredom. The day starts very early, and apart from brief diversions such as meals, ward rounds, examinations, therapies, or visits, there are few distractions. People with cognitive impairments often find it difficult to keep themselves occupied.

Reasons for these changes can be

- Loss of skills (vision, hearing, dexterity)
- Loss of memory
- Lack of drive

Keeping busy is one of the most important activities in a person's life. Occupation is meaningful, can create joie de vivre, reduce restlessness, and promote existing skills.

The main features of the engagements are:

- The engagement should be fun
- The duration should be limited to 15-20 minutes
- At the first signs of fatigue, disappointment, or rejection, the engagement should be terminated
- The tasks must be achievable
- The aim is to create a "sense of achievement", by offering support if needed
- Offer something to drink regularly during the activity

Engagement opportunities must be selected carefully, especially in a hospital setting. The effort and preparation time must be kept to a minimum and the engagement must be easy to implement. Examples of engagement opportunities are:

- Playing board games e.g. Memory, Dominoes, Categories, Ludo, crossword puzzles
- Reading, reading stories aloud or listening (poems, daily newspaper)
- Using a virtual reality headset for activation with different scenarios
- Listening to music, singing together - ask for their favorite music
- Looking at pictures/photo albums to bring back memories
- If necessary, assistance with dressing and personal hygiene, eating, or going to the toilet

Music is considered the "silver bullet" for accessing people with cognitive impairments or post-operative delirium. Singing together is generally very well received. CDs can also be played for people to sing along to. Suitable songs for older people are:

- Folk songs
- Pop songs from the patients' youth
- Well-known operetta melodies
- Christmas carols (depending on the season)
- Hymns

Although singing very often has positive effects, there are also older people who do not like singing or do not sing at all. As with all activities, it is necessary to closely observe how patients react.

2.4 FIT Visit

Implementation: Physiotherapists and sports scientists.

Objectives: Many older patients think that when they are ill, they must stay in bed until they feel better again. However, various functional disorders of the body's organs occur during prolonged bed rest. Within a few days, immobility leads to a deterioration in general health, loss of muscle strength, and balance problems. Other health risks include the risk of pneumonia or venous thrombosis, as well as the occurrence of skin issues. Many of these problems can be avoided if older people exercise regularly, get up every now and then, and walk a few steps.

Execution: The aim of the "Fit Visit" is to keep older people moving during their stay in the hospital. Patients who can stand up should be given help and assistance with walking twice a day through the so-called "Fit Visit". For patients who are unable to walk for health reasons, there are instructions on how to perform simple movement exercises in bed (**Table 2**).

Table 2 Movements in bed

Movements in bed

1. Raise your arms - inhale
   Lower your arms - exhale
   Raise your left shoulder - inhale
   Lower your shoulder - exhale
   Raise your right shoulder - inhale
   Lower your shoulder - exhale
2. Tilt your head back - inhale
   Tilt your head forward - exhale
   Turn your head to the left - inhale
   Turn your head to the front - exhale
   Turn your head to the right - inhale
   Turn your head to the front - exhale
3. Tilt your upper body to the right - inhale - exhale
   Return your upper body to the middle - inhale - exhale
   Do the same to the left side
4. Inhale - bend your upper body forward - exhale
   Straighten your upper body – inhale
5. Turn your upper body to the right and left - inhale - exhale

Table 3 Notes on exercises from a sitting and lying position

Standing Up from a Sitting Position:

- Support the natural movement sequence.
- Both legs are hip width apart, one foot positioned slightly behind the other.
- Encourage active participation by having the person push up using the armrests of the chair.
- Stand next to the person and assist the movement by gently placing your hands on the person's knee and lower back.

Getting Up from a Lying Position:

- The person begins in a side-lying position and swings both legs over the edge of the bed, one after the other. Using the forearm that is underneath, the person pushes off from the mattress. Stand at head level and assist this motion by placing your arm along the person's shoulder to provide support.

If a Walking Aid is Used, Walk on the Opposing side.

- Place your arm under the arm of the person being assisted, as if you were linking arms.
- Your upper arm should be positioned between the person's chest and upper arm; your forearm supports their forearm.
- Place your arm that is closest to the person around their waist at hip level. Use your opposite arm to support their forearm.

Important: Never grip or guide the person by their upper arm, and never pull them by placing your hands under their armpits.

2.5 Assistance with meals

Implementation: Research assistants and treatment assistants.

Objectives: Maintaining good nutrition in older people and people with cognitive impairment is a particular challenge. Good nutrition promotes the healing process and strengthens the natural defenses against infections.

Execution: An adequate supply of food and drink can also help to prevent confusion. There are many reasons why older patients may find it difficult to maintain a good nutritional status. The "AKTIVER Heart" team can provide various types of assistance with eating and drinking, depending on the patient's needs and abilities.

In detail, these are:

- Helping with the menus
- Company at mealtimes
- Encouraging eating and drinking
- Assistance with food intake
- Documenting food and fluid intake

Appetite and thirst diminish with age. Disturbances in fluid and food intake are therefore common, especially if other conditions restrict food intake.

In the event of abnormalities, a nutritional therapist is informed and consulted at a low-threshold level.

Possible causes:

- Reduced sense of smell and taste
- Visual impairment
- The situation is misjudged (eating in bed!)
- Decreased production of digestive juices
- Missing teeth
- inflammation in the mouth
- Slower digestion
- Diseases e.g. diabetes
- Restricted ability to move
- Unfamiliar food
- Unfamiliar surroundings and people
- Loneliness
- Distractions
- Medication

Tips for stimulating the appetite

- Request favorite food
- Small, nicely arranged portions
- more seasoning, if necessary (herbs! / be careful with salt)
- Sweeten drinks / food if necessary
- Company, possibly join in the meal

Assistance with eating

- Sit with good posture
- Cut food into small pieces, open packaging
- Correct temperature control for drinks / food
- Create an order on the tray
- Possibly offer finger food
- Allow time for food intake
- Reduce disturbances

2.6 Relaxation and sleep promotion

Implementation: Research assistants and treatment assistants.

Objectives: Sleeping disorders in hospital patients are a significant problem and can sometimes have considerable consequences. Adequate sleep is crucial for recovery from illness. The "normal" sleep rhythm follows physiological rules and is important for restful sleep. In the hospital, there is an increased risk of this rhythm being disrupted by hospital procedures, as wake-up and sleep times are based on the needs of the hospital. The nights are "too long". Almost all patients are affected by this!

Other possible causes of sleep disorders in hospital can be:

- Unfamiliar surroundings
- Sounds, noise
- Restlessness and fear

Execution: In older people, these changes can lead to disorientation or a "day-night-reversal". To fall asleep well in the hospital, it is not enough to simply switch off the light or take a sleeping pill. Measures that help the patient to rest in the evening can improve the healing process and help to prevent unwanted complications (restlessness at night, wandering, falls, delirium).

Examples:

- Pleasant, calm music
- Offer a warm drink (milk, sleeping tea)
- Ensure a relaxed sleeping atmosphere (ventilation, correct room temperature)
- Aromatic wipes (lavender oil, orange blossom oil, rose oil)
- Reduce sources of noise
- Additional pillows / blankets
- Sleep ritual

Table 4 Exercises to promote sleep

Exercise: Promotion of Sleep

(The Hospital Elder Life Program© 2000, Sharon K. Inouye, MD, MPH)

Preparation: Wash your hands.

- Knock on the door, address the patient respectfully (Mr./Mrs.)
- Introduce yourself, explain your role.

Start the conversation:

- I would like to help you have a good night's sleep.
- Smile, eyes: positive contact.
- Voice: slow and calm, not too loud.
- Movements: calm, relaxed.

Adjustments of the room promote better sleep

- Reduce noise: ask for permission turn off the TV. Close the curtains and door; ask neighbors for support.
- Give the patient the opportunity to use the toilet.
- Ensure that the patient is lying comfortably.
- Pleasant room temperature
- Lighting: Switch off or dim all lamps

Give the patient the opportunity to use the toilet.

- Offer and serve herbal tea / warm milk
- Offer relaxing music (ask the patient what they like)
- Offer warm lotion for the hands
- Offer a back massage (Enclosure: Instructions for back

Wishing you a "good night" - leave the room quietly

Table 5 Relaxation exercises

Exercise: Relaxation

(The Hospital Elder Life Program© 2000, Sharon K. Inouye, MD, MPH)

Preparation: Wash your hands.

- Knock on the door, address the patient as "you" (Mr., Mrs. ...).
- Introduce yourself, explain your role.

Start the conversation:

- "I'm here to do relaxation exercises with you" or "I have a relaxation exercise here that you can learn and do on your own".
- Smile, eyes: positive contact.
- Voice: slow and calm, NOT monotonous.
- Movements: calm, relaxed.

Ensure the patient's comfort:

- He should sit or lie comfortably to avoid interruptions
- Lighting switched off
- Pleasant room temperature

Turn on some relaxing music (CD player or Walkman).

- Ask the patient to think of a desired place. Help the patient to remember a place where they were most relaxed; ask for a brief description later.

Determine breathing rhythm:

- Breathe in slowly through your nose
- Hold your breath ... two ... three ...
- Breathe out completely through the mouth.

2.7 Accompaniment to diagnostic appointments

Implementation: Research assistants and treatment assistants of the AKTIVER Heart team.

Objectives: The numerous examinations before and after heart surgery can lead to a considerable overload for older patients. These include, for example, chest X-rays, pulmonary function diagnostics, CT examinations of the chest, echocardiography, and transcranial doppler ultrasound of the cerebral vessels if necessary. Additional examinations such as a CT of the skull, dental consultations (especially before planned heart valve surgery, if not already performed) and supplementary examinations may also be necessary. In addition, patients often must wait up to 30 minutes (sometimes up to 60 minutes) for patient transport services within the University Medical Center Göttingen. This waiting time can also lead to physical stress for older, often frail patients (hypothermia, local disorientation, hypoglycemia, exsiccosis, etc.).

Execution: The AKTIVER Heart Team will accompany the intervention patients to the planned/elective examinations before and after the heart operation (if time permits). The accompaniment will not replace the patient transportation service but merely help with orientation. In addition, stressors (e.g. cold, thirst, etc.) can be reduced with the help of the AKTIVER Heart staff.

The following aspects can be applied during the accompaniment:

- Discussion of the contents of the Flower of Life with a focus on resource activation
- Providing clothing
- Providing drinks or snacks
- Education about the necessity of the examination
- Calming the patient during stress due to the numerous examinations
- Playing of music
- If necessary, arrange patient transport by the hospital's transport service

3. Cognitive, psychological and behavioral changes

3.1 Challenging behavior

Behavior perceived as disruptive, distressing, and dangerous to others or oneself is considered challenging. The term "challenging behavior" refers solely to its impact on the environment, not how the person experiencing it interprets their own actions.

What caregivers find challenging may make sense to the patient or even support their well-being. Therefore, if a behavior enhances the patient's quality of life and causes no harm to others, caregivers should tolerate it.

Behavior often perceived as stressful by caregivers:

- Aggressiveness
- Restlessness or agitation
- Vocal disturbances (e.g. frequent shouting, screaming, or making noises)
- Aimless wandering
- Apathy or withdrawal

Other challenging behaviors in the context of pre-existing cognitive dysfunction or delirium are: frequent verbal abuse or accusations, clinging or distressing behavior (possibly also sexually distressing behavior), frequently leaving the home when disoriented, removal of incontinence pads combined with defecating and urinating in the home, frequent nocturnal restlessness, vomiting, destroying furnishings or frequently putting things away, endangering themselves and/or others when handling fire, stove, household objects or in traffic, eating spoiled or inedible food, constant whining and complaining, severe anxiety or delusional fears. Withdrawal and apathy are often perceived as far less stressful and therefore less noticeable than active disorders such as aggression or constant shouting. However, apathy and withdrawal can be more stressful for the patient than obviously disruptive behavior. Caregivers should therefore not limit their attention exclusively to highly disruptive behavior.

The primary aim in managing challenging behavior should always be to improve the well-being of the patient (and, if necessary, their relatives), not merely to suppress their behavior. Achieving this requires an understanding of its triggers and context. It is necessary to document the patient's mental state, i.e. how they experience their own behavior, and record precise descriptions of the situation, including verbal expressions and body language. The NDB model (need driven dementia compromised behavior model), for example, helps to conceptualize these behaviors. It distinguishes between background factors that are hard to modify, e.g. those resulting from the direct consequences of the illness (cognitive changes, health status, etc.), and proximal factors that can be influenced (environmental conditions, current needs, etc.).

Conditions for challenging behavior according to the NDB model

In many cases, it can be assumed that challenging behavior is an attempt to adapt, express, communicate, make sense of situations, and assert oneself in relation to the environment on a different level of psychological functioning. Certain behaviors such as living in the past, clinging (behaviors, apathy, and complete withdrawal serve the desire to cope with powerful feelings such as fear, sadness, and despair and to protect oneself. Behavior is highly variable regarding different situations: As a rule, it is more the context that generates behavior, rather than a consideration or decision by the sick person or the inherent dynamics of the illnesses. Conversely, a differentiated knowledge of psychiatric symptoms, the psychopathological findings, and their etiology is required in order not to overestimate the psychosocial component and to avoid the danger of working in vain towards unattainable goals.

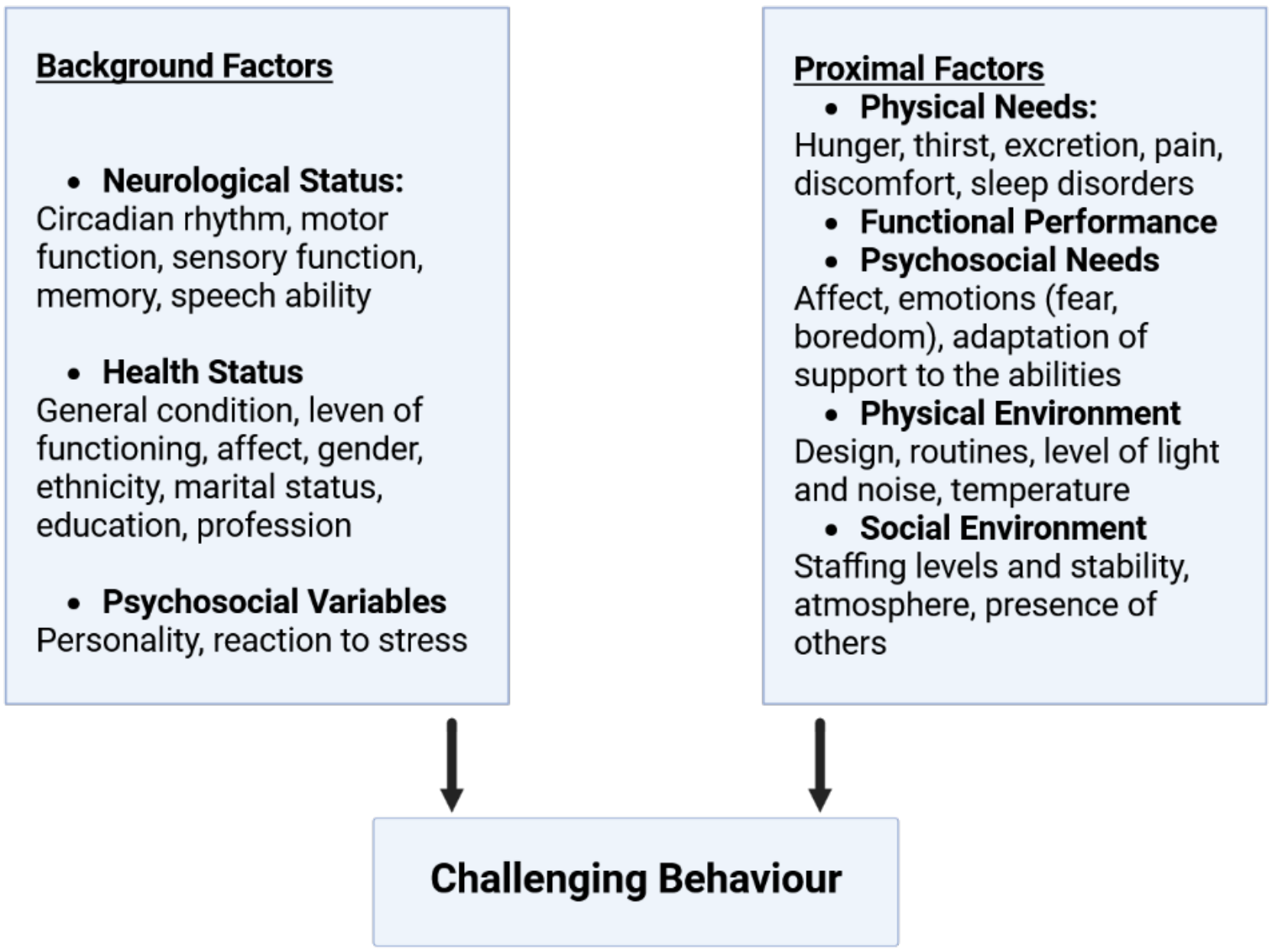

Figure 6 Developmental model of challenging behavior (Bartholomeyczik, 2013)

An approach to aggressive tendencies considers the connection between underlying agitation and stimulus response. Like the NDB model, general factors set the basic arousal level, while situational factors, when they exceed a threshold value, trigger anger reactions or aggressive behavior [1]

4. Communication and conversation

Objectives of this unit: To raise awareness of the subjectivity of perception, to reflect on one's own conversational behavior, and thus become more mindful of it. By studying changes in communication in old age and people with cognitive dysfunction or postoperative delirium, difficult conversational situations can be anticipated and possible solutions developed.

- What do I know about my speaking behavior?
- How well can I listen?
- Am I easily distracted during conversation?
- How do external circumstances influence me (e.g. hectic everyday life, restlessness, noise)?
- How do I react to my counterpart's expressions of emotion?
- Can I perceive implicit statements?
- Are there any difficult topics that I "fend off"?
- Are there any taboo subjects for me?
- What happens if I like / dislike someone?
- What if someone triggers strong feelings in me?

4.1 Multi-layered communication

Paul Watzlawick set out 5 basic rules (pragmatic axioms) that explain human communication and reveal its paradoxes:

1. One cannot not communicate
2. Every communication has a content and a relationship aspect
3. The nature of a relationship is dependent on the punctuation of the communicational sequences between the communicants
4. Human communication involves both digital and analog modalities
5. Inter-human communication procedures are either symmetric or complementary

Every message is significantly influenced by its relationship aspect, which determines how the message is understood by both conversation partners. No content can be expressed without the relationship aspect.

Example: "My right arm hurts" can mean:

- ....
- ....
- ....

Messages/statements have meanings that need to be understood. Misunderstanding is the default state in communication. The following quote illustrates this:

"What is thought is not always said; what is said is not always heard; what is heard is not always understood; what is understood is not always agreed; what is agreed is not always applied; what is applied is not always retained."

4.2 Verbal and non-verbal communication

Non-verbal signals account for around 80 percent of a message's impact. It is therefore very important how something is communicated. Facial expressions and gestures are largely the result of a socialization process and therefore vary by culture. For example, the Japanese use their faces less than Germans do and Italians use their hands more than Germans do.

4.3 Communication in cases of delirium or cognitive dysfunction

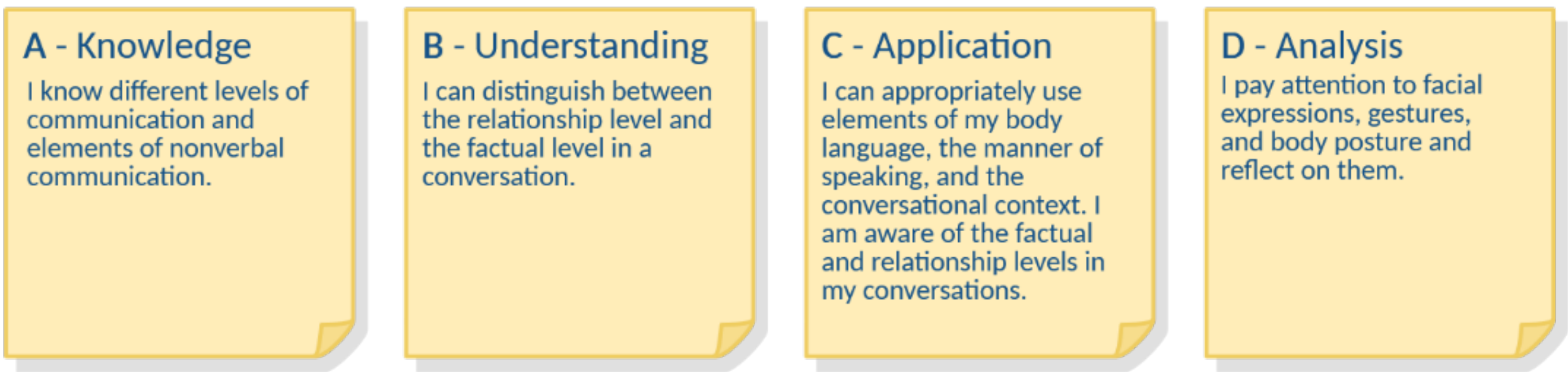

Figure 7 Aspects of verbal and non-verbal communication

When communicating/interacting with people experiencing cognitive dysfunction, the aim should be to reassure them that they are understood. As this is not always possible, a communication style is needed that conveys empathy and clarity. The following principles should be considered:

- Approach from the front, make eye contact, and introduce yourself
- Address your counterpart by name regularly
- Speak slowly (max. 2 words / second), calmly, and with a friendly facial expression
- Use short, simple, and familiar words
- Convey only one message at a time
- Ask only one question at a time
- Supplement the spoken word with gestures and tactful touches
- Show that you are listening and trying to understand what is being said (excursus: active listening)
- Do not interrupt! Avoid arguing or criticizing

When communicating with people with cognitive dysfunction/postoperative delirium, it is important to focus on positive things. Often those affected are unable to finish a sentence. We must then ensure that communication is successful. Ways to achieve this are

- Make positive suggestions - e.g. Let's go ...
- Make suggestions if the person is having trouble deciding
- Be understanding and patient. Physical contact. Pay attention if the person does not want this!
- Give compliments!
- Talk about current affairs (hairstyle, clothing, etc.)
- Talk about the past. These topics should have a positive connotation (biographical knowledge)

4.4 Validation and Integrative Validation according to Richard®

Validation is both a communication method and a therapeutic approach for connecting with people who suffer from forgetfulness and disorientation. It helps the confused elderly person to regain their dignity. Validation means accepting the disoriented person living in the past as they are, showing them respect and empathy. The validation worker accepts the elderly person and empathizes with them, trying to "walk a mile in the other person's shoes" (cf. Feil, 2016)

The primary aims of validation are to restore self-esteem and reduce stress.

Principles of validation:

- Validated feelings lose their intensity (Freud, Jung)
- Ignored feelings gain intensity
- Early, consolidated emotional memories are retained (Skinner, Watson)
- As short-term memory deteriorates, earlier memories resurface
- We can all see and hear with our souls (Penfield). Validation is a method with 15 verbal and non-verbal techniques

Integrative Validation (IVA, "to declare something valid, to value") was developed by Nicole Richard (1957-2014), a qualified educationalist and psycho-gerontologist. Integrative Validation differs from Validation according to N. Feil mainly in that it does not aim to overcome unresolved life problems. IVA is an approach and an attitude that focuses on the feelings and driving forces of the patient with cognitive dysfunction/postoperative delirium. Their perceived world and the patient's view of reality take center stage. The focus is no longer on the present, but the caregivers try to empathize with the "time and experience level" of the confused person. The feelings and driving forces of the confused person are recognized, accepted, and valued. The emotions of a

confused person are real. They must not be rejected by their environment. The patient's expressions are not corrected, and the confused person is not confronted with their failures.

The feelings are accepted instead of being taken away, talked away, toned down, or even ridiculed. No explanations are sought. The person with dementia acts this way because they have dementia. We don't think about what the person with dementia is doing, but how they are doing it and what the motivation behind it is.

Through Integrative Validation, the confused person feels increasingly accepted and gains confidence and security: **"Someone understands me."**

1. The feeling of **sensing** the patient's drive
2. **Validate,** i.e. accept, appreciate and value feelings and drives using short, direct sentences
3. **Validate in general**, i.e. react to what is said with proverbs, folk wisdom

Example: "You stole money from me"

1. **Feelings and drives are:** Anger, rage, annoyance, indignation, distrust, despair, fear
2. **Validation:** "You are angry". "That would make anyone's blood boil". "You must be seething with anger". "Who can you trust anymore?". "It's enough to make one despair". "You feel completely helpless". "It can be frightening".
3. **General validation:** "Be careful whom you trust". "What is right must remain right".

Options for Validation:

Reacting to anger:	My goodness, you sound so upset!
Sadness:	You seem very sad; you have a heavy heart.
Desperation:	You don't know what to do, do you?
Honesty:	You are an honest person!
Diligence:	You are a hard worker! You are always on the move!
Duty:	You have a strong sense of duty
Punctuality:	I could set my watch by you, punctual as you are.

1. Bartholomeyczik, S., Halek, M., & Holle, D. (2013). *Herausforderndes Verhalten bei Menschen mit Demenz verstehen: Die Verbesserung der Versorgung Demenzkranker durch Qualitätsinstrumente*. Weinheim: Beltz.
