# Supplementary material for: Feasibility, acceptability, and preliminary estimates of a multimodal AKTIVER Heart Delirium Prevention Program in patients indicated for cardiac surgery: study protocol of a randomized controlled PREVENT POstoperative Delirium (PREVENT-POD) Pilot Study
Source: Pilot Feasibility Stud. 2026 Oct 5;12:142. doi: 10.1186/s40814-026-01923-6 (PMC13637325; doi:10.1186/s40814-026-01923-6)
Supplement: Supplementary file 2 [file 40814_2026_1923_MOESM2_ESM.pdf]

RESPIRATORY THERAPY

Objective: Develop breathing awareness, learn pursed lip breathing, strengthen respiratory muscles.

Incentive Spirometer "Coach 2": You will receive the incentive spirometer "Coach 2" before your surgery. Use it to carry out **10 breathing cycles 6 times a day** as a self-training exercise.

Instructions: Breathe in through the mouthpiece (see Fig. 1a), keeping the yellow indicator inside the area marked with a smiley face (see Fig. 1b). Then exhale slowly while pursing your lips. The resulting resistance extends the period of exhalation. Take a break of up to two minutes after each breathing cycle (inhalation and exhalation).

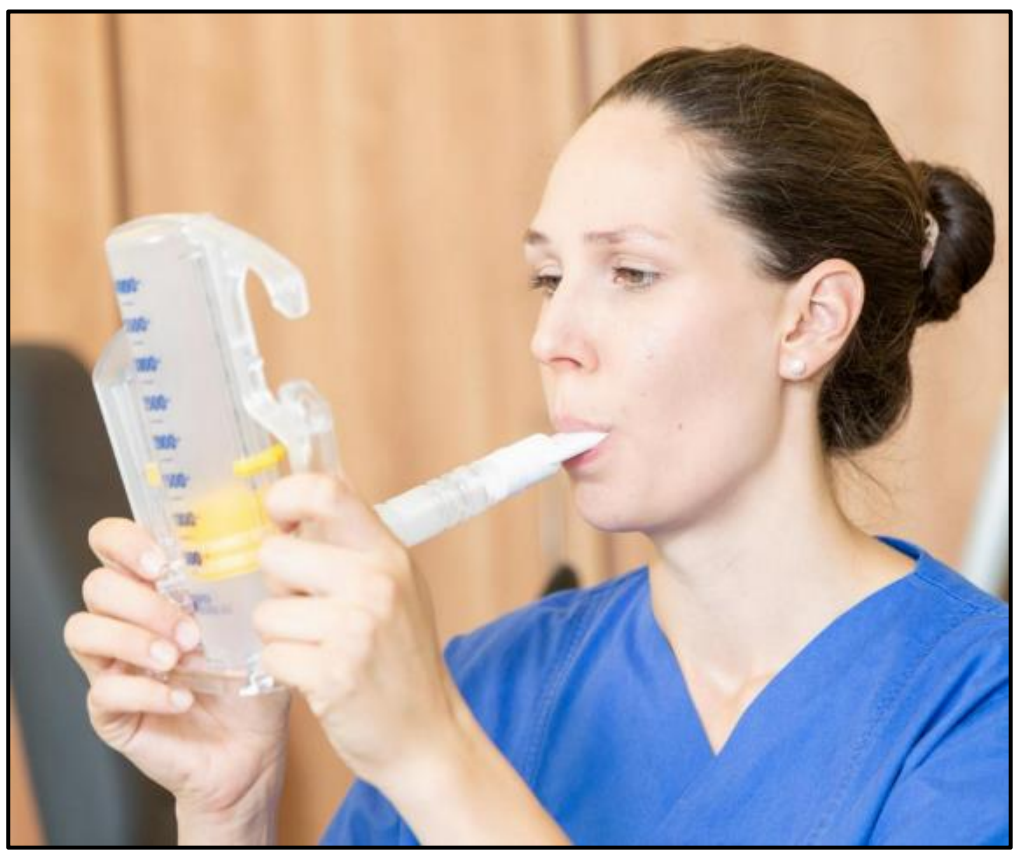

Fig. 1a: Illustration of the use of "Coach 2"

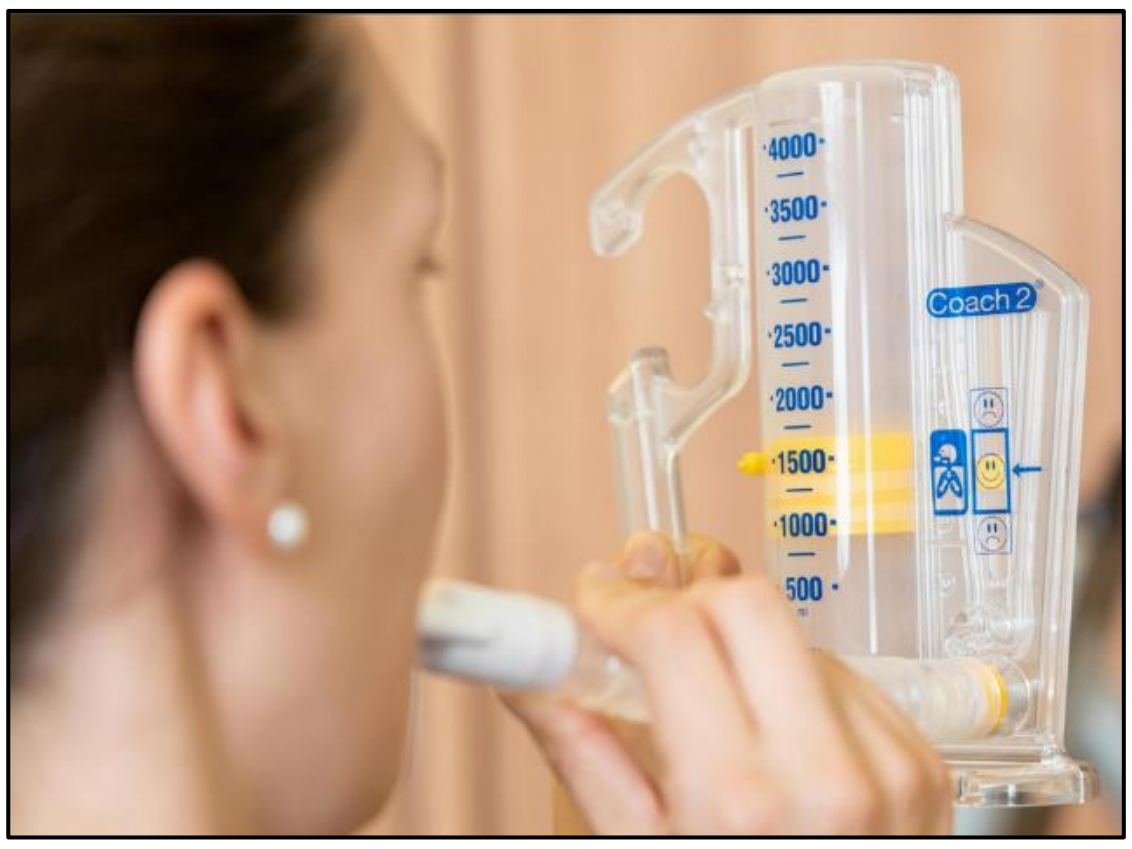

Fig. 1b: Target position for inhalation; yellow indicator at the height of the smiley face

Caution: Ensure that the intensity of endurance and strength training remains within your individual symptom tolerance. If you experience symptoms such as radiating pain in the arm or chest area, immediately reduce the intensity of the activity and take a break.

SPORTS/MOVEMENT THERAPY

Endurance Exercise

Objective: Increase physical endurance, improve body awareness, enhance quality of life.

Instructions: Carry out light endurance training **3 times a week for 10 to 60 minutes** by taking a walk on flat terrain. The intensity of the exercise should allow you to hold a conversation without difficulty.

Strength Exercises

Objective: Build muscular strength and support everyday activities.

Exercise 1: Sit upright and simulate walking movements with your legs for **one minute**, ensuring that your breathing remains steady and unimpeded (see Fig. 2a). Position a watch or timer with a clearly visible second hand in front of you for timing.

Exercise 2: Extend both arms straight in front of your body. Alternately open and close your hands for one minute (see Fig. 2b).

Exercise 3: Sit upright and extend your legs alternately straight out, performing 3 sets of 10 repetitions per leg. Allow for a rest period of up to one minute between sets (see Fig. 2c).

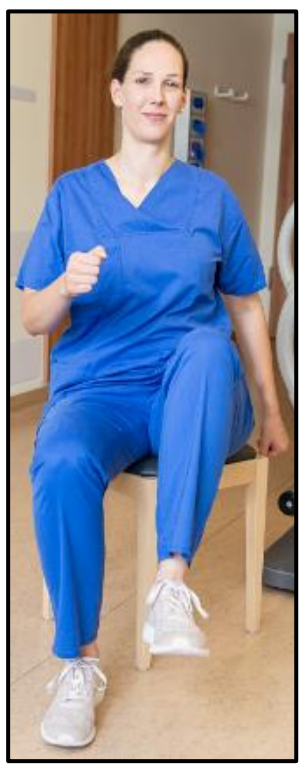

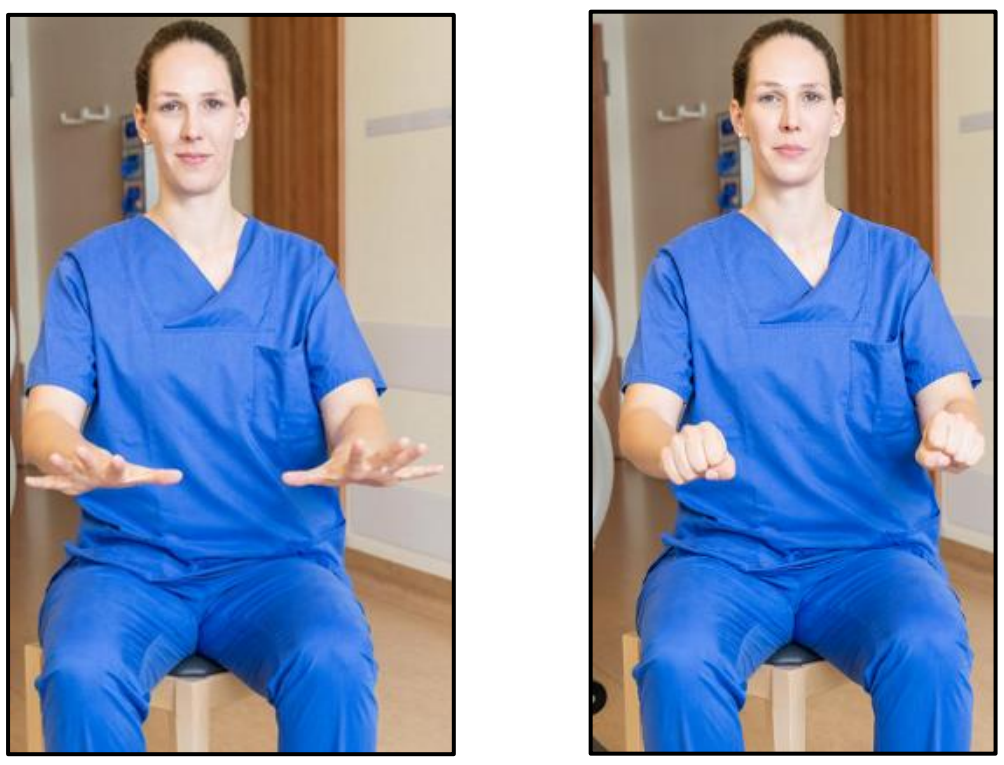

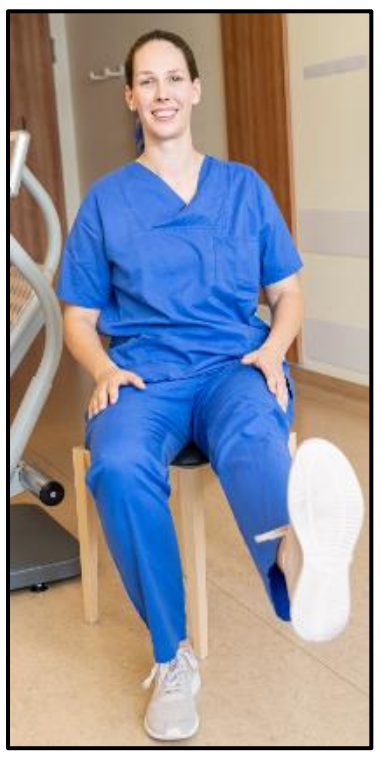

Fig. 2a: Exercise 1 **Fig. 2b**: Exercise 2 starting and end position **Fig. 2c**: Exercise 3

Caution: Ensure that the intensity of endurance and strength training remains within your individual symptom tolerance. If you experience symptoms such as radiating pain in the arm or chest area, immediately reduce the intensity of the activity and take a break.
