# Supplementary material for: Feasibility, acceptability, and preliminary estimates of a multimodal AKTIVER Heart Delirium Prevention Program in patients indicated for cardiac surgery: study protocol of a randomized controlled PREVENT POstoperative Delirium (PREVENT-POD) Pilot Study
Source: Pilot Feasibility Stud. 2026 Oct 5;12:142. doi: 10.1186/s40814-026-01923-6 (PMC13637325; doi:10.1186/s40814-026-01923-6)
Supplement: Supplementary file 4 [file 40814_2026_1923_MOESM4_ESM.pdf]

UNIVERSITÄTSMEDIZIN GÖTTINGEN UMG

Ethik-Kommission der Universitätsmedizin Göttingen, Von-Siebold-Straße 3, 37075 Göttingen

Ethik-Kommission der
Universitätsmedizin Göttingen
Vorsitzender: Prof. Dr. Jürgen Brockmöller
Referentin
Regierungsrätin Doris Wettschereck
0551 / 39-68644 **Telefon**

Von-Siebold-Straße 3, 37075 Göttingen
Adresse
0551 / 39-61261 **Telefon**
0551 / 39-69536 **Fax**
ethik@med.uni-goettingen.de **E-Mail**
www.ethikkommission.med.uni-goettingen.de

Frau
Dr. med. Monika Sadlonova
Klinik für Thorax-, Herz- und Gefäßchirurgie
Robert-Koch-Straße 40
37075 Göttingen

per E-Mail: Monika.Sadlonova@med.uni-goettingen.de

30.10.2024 br – fr - gö Datum

**Antragsnummer:**	**15/9/24 (bitte stets angeben)**
**Studientitel:**	Machbarkeit, Akzeptanz und vorläufige Wirksamkeit eines multimodalen AKTIVER Heart Delir-Präventionsprogramms bei Patient*innen mit Indikation zu einem herzchirurgischen Eingriff: Randomisiert kontrollierte PREVENT POstoperative Delirium (PREVENT-POD) Pilotstudie
**Antragsteller:**	Dr. med. Monika Sadlonova, Prof. Dr. med. Ingo Kutschka, Julia Kühnle, Prof. Dr. med. Hassina Baraki, PD Dr. med. Aschraf El-Essawi, Dr. med. Fawad Jebran, PD Dr. med. Ruben Evertz, Klinik für Thorax-, Herz- und Gefäßchirurgie, Prof. Dr. med. Christine von Arnim, Dr. med. Carolin Steinmetz, Klinik für Geriatrie, Prof. Dr. med. Christoph Herrmann-Lingen, Klinik für Psychosomatische Medizin und Psychotherapie, Dr. Marcus Wuttke, Abt. für Physiotherapie, UMG<br>Doktorand*in: Minh Tuan Dong

Folgende Unterlagen wurden zur Bewertung nachgereicht:

- Anschreiben vom 13.10.2024 (E-Mail)
- Stellungnahmen zu Schreiben der EK vom 27.09.2024 mit Schreiben vom 13.10.2024
- PREVENT-POD Ethikantrag/Studienprotokoll, Version 2.0 vom 04.10.2024, mit markierten Änderungen
- PREVENT-POD Patienteninformation und -einwilligung, Version 2.0 vom 04.10.2024, mit markierten Änderungen
- PREVENT-POD Erhebungsinstrumente T0-T4, Version 1.0 vom 28.08.2024
- AKTIVER Heart Interventions- und Schulungsmanual (Kurzform), Version 1.0 vom 28.08.2024
- AKTIVER Heart Informationsmaterial für Ausdauer- und Krafttraining sowie Atemtherapie, Version 1.0 vom 04.10.2024
- Flyer der PREVENT-POD Studie für den Rekrutierungsprozess, Version 1.0 vom 28.08.2024
- Lebenslauf Dr. med. Monika Sadlonova inkl. GCP-Zertifikate (Studienleiterin) vom 28.08.2024
- Lebenslauf Prof. Dr. med. Christine von Arnim (stellvertretende Studienleiterin) vom 28.08.2024
- Bestätigung des Klinikdirektors für Psychosomatische Medizin und Psychotherapie, Herrn Prof. Dr. Christoph Herrmann-Lingen vom 08.10.2024

Sehr geehrter Frau Dr. Sadlonova, sehr geehrte Damen und Herren,

nach Ergänzung der o.g. Dokumente und Beantwortung der im vorläufigen Votum aufgeführten Fragen in ihrem Schreiben vom 13.10.2024 bestehen nunmehr keine ethischen und rechtlichen Bedenken gegen die Durchführung des oben genannten Forschungsvorhabens.

Bitte beachten Sie noch folgenden Hinweis:
Wir gehen davon aus, dass die Beteiligung an der Durchführung der Studie von den Leitern der Abteilung Physiotherapie genehmigt wurde.

Es liegt in der Verantwortung der Antragsteller, die oben angeführten Hinweise vollständig umzusetzen. Ein weiteres Votum ergeht nicht.

Wir wünschen Ihnen viel Erfolg bei der Durchführung Ihres Projektes.

Unabhängig vom Beratungsergebnis macht die Ethik-Kommission darauf aufmerksam, dass die ethische und rechtliche Verantwortung für die Durchführung einer wissenschaftlichen Studie beim verantwortlichen Studienarzt und aller an der Studie beteiligten Ärzte liegt.

Alle Änderungen im Studienprotokoll müssen der Ethik-Kommission vorgelegt werden und dürfen erst nach deren Beratung und Bewertung umgesetzt werden.

Über alle schwerwiegenden unerwarteten unerwünschten Ereignisse, die während der Studie auftreten und die Sicherheit der Studienteilnehmer oder die Durchführung der Studie beeinträchtigen könnten, muss die Ethik-Kommission unterrichtet werden.

Der Abschluss/Abbruch der Studie ist mitzuteilen und ein Abschlussbericht vorzulegen.

Auf die Einhaltung einschlägiger Gesetze und Rechtsvorschriften wird hingewiesen. Die nach Rechtslage notwendigen Unterrichtungen (u. A. Änderung des Studienprotokolls, Meldung von Zwischenfällen, neue Datenlage, Nachmeldung von Prüfzentren, Abschlussbericht) sind der Ethik-Kommission unverzüglich vorzulegen.

Die Ethik-Kommission bestätigt, dass sie auf Grundlage nationaler Gesetze, Vorschriften sowie der GCP/ICH-Richtlinie arbeitet.

Hinweise zu den datenschutzrechtlichen Aspekten:
Details zu Ihren Informationspflichten gegenüber den Studienteilnehmern entnehmen Sie bitte insbesondere den Art. 13 ff DSGVO. Die Ethikkommission prüft die Angaben zu den zuständigen Datenschutzbeauftragten und Aufsichtsbehörden nicht auf Richtigkeit.
Datenschutzrechtliche Aspekte von Forschungsvorhaben werden durch die Ethikkommission grundsätzlich nur kursorisch geprüft. Dieses Votum / diese Bewertung ersetzt mithin nicht die Konsultation des zuständigen Datenschutzbeauftragten.

Wir weisen darauf hin, dass Sie nach der Deklaration von Helsinki (Nr. 35) verpflichtet sind, Ihre Studie vor Beginn bei einem zur *International Clinical Trials Registry Platform (ICTRP)* gehörendem Studienregister zu registrieren.

Es wird bestätigt, dass verantwortliche Studienärzte, die an der o.g. Studie beteiligt sind, nicht an der Beratung und Abstimmung teilgenommen haben.

Mit freundlichen Grüßen

Prof. Dr. med. J. Brockmöller
Vorsitzender der Ethik-Kommission

UNIVERSITÄTSMEDIZIN GÖTTINGEN UMG

Ethics Committee of
University Medical Center
Göttingen Chairman: Prof. Dr. Jürgen
Brockmöller
Speaker
Government Councillor Doris Wettschereck
0551 / 39-68644 **Phone**

Von-Siebold-Straße 3, 37075 Göttingen
Address
0551 / 39-61261 Phone
0551 / 39-69536 Fax
ethik@med. uni-goettingen.de E-Mail
www.ethikkommission.med.uni-goettingen.de

Woman
Dr. med. Monika Sadlonova
Department for Thoracic, Cardiac and Vascular Surgery
Robert-Koch-Straße 40
37075 Göttingen

by e-mail: Monika.Sadlonova@med.uni-goettingen.de

30.10.2024 br - fr - gö Date

Application no:	15/9/24 (please always specify)
Study title:	Feasibility, acceptability and preliminary efficacy of a multimodal ACTIVE Heart Delirium prevention program in patients with an indication for cardiac surgery: Randomized controlled PREVENT POstoperalive Delirium (PREVENT-POD) pilot study
Applicants:	Dr. med Monika Sadlonov a, Prof Dr med Ingo Kutschka , Julia Kühnle, Prof Dr med Hassina Baraki, PD Dr med Aschraf El-Essawi, Dr med Fawad Jebran , PD Dr med Ruben Evertz, Clinic for Thoracic, Cardiac and Vascular Surgery, Prof. Dr med Christine von Arnim, Dr med Carolin Steinetz, Clinic for Geriatrics, Prof. Dr med Christoph Herrmann Lingen, Clinic for Psychos omal Medicine and Psychotherapy Lingen, Clinic for Psychos omal Medicine and Psychotherapy Lingen, Clinic for Psychos omal Medicine and Psychotherapy Dr. med. Christine von Arnim, Dr. med. Carolin Steinmetz, Clinic for Geriatrics, Prof. Dr. med. Christoph Herrmann Lingen, Clinic for Psychos omal Medicine and Psychotherapy, Dr. Marcus Wutlk e, Department of Physiotherapy, UMG
	Doctoral candidate: Minh Tuan Dong The

following documents were subsequently submitted for evaluation:

- Cover letter dated 13.10.2024 (e-mail)
- Comments on theEK's letter dated 27.09.2024 with|letter dated 13.10.2024
- PREVENT-POD ethics application/study protocol, version 2.0 dated 04.10.2024, with marked changes
- PREVENT-POD patient information and consent, version 2.0 dated 04.10.2024, with marked changes
- PREVENT-POD survey instruments T0-T4, version 1 0 from 28.0B.2024
- AKTIVER Heart intervention and training manual (short form), version 1.0 dated 28.08.2024
- AKTIVER Heart information material for endurance and strength training and respiratory therapy, version 1.0 dated 04.10.2024
- Flyer of the PREVENT-POD study for the recruitment process, version 1.0 from 28.08.2024
- Curriculum vitae Dr. med. Monika Sadlonova incl. GCP certificates (principal investigator) dated 28.08.2024 Curriculum vitae Prof. Dr. med. Christine von Arnim (deputy principal investigator) dated 28.08.2024
- Confirmation of the Clinic Director for Psychosomatic Medicine and Psychotherapy, Prof. Dr. Christoph Herrman n- Lingen dated 08.10.2024

Dear Dr. Sadlonova, ladies and gentlemen.

After reviewing the above-mentioned documents and answering the questions listed in the preliminary vote in your letter dated October 13, 2024, there are now no ethical or legal objections to the implementation of the above-mentioned research project.

Please note the following information:
We assume that participation in the study has been approved by the heads of the physiotherapy department.

It is the responsibility of the applicant to implement the above instructions in full.

We wish you every success with the implementation of your project.

Irrespective of the outcome of the consultation, the Ethics Committee would like to point out that the ethical and legal responsibility for conducting a scientific study lies with the responsible study physician and all physicians involved in the study.

All changes to the study protocol must be to the Ethics Committee and may only be implemented after its consultation and assessment.

The Ethics Committee must be informed of any serious unexpected adverse events that occur during the study and could affect the safety of the study participants or the conduct of the study.

The completion/termination of the study must be communicated and a final report submitted.

Attention is drawn to compliance with relevant laws and regulations. The information required by law (including changes to the study , notification of adverse events, new data situation, subsequent notification of trial centers, final report) must be submitted to the Ethics Committee without delay.

The Ethics Committee confirms that it works on the basis of national laws, regulations and the GCP/ICH guideline.

Notes on data protection aspects:
For details on your information obligations towards the study participants, please refer in particular to Art. 13 ff GDPR. The Ethics Commission does not check the information on the responsible data protection officers and supervisory authorities for accuracy. Data protection aspects of research projects are generally only examined cursorily by the Ethics Committee. This vote/assessment9 therefore does not replace consultation with the responsible data protection officer.

We would like to point out that according to the Declaration of Helsinki (No. 35) you are obliged to register your trial with a trial registry belonging to the *Clinical Trials Registry Platform (ICTRP)* before the start of the trial.

It is confirmed that responsible study physicians involved the above-mentioned study did not participate in the consultation and voting.

Mit freundlichen Grüßen

Prof. Dr. med. J. Brockmöller
Vorsitzender der Ethik-Kommission
