# Supplementary material for: Feasibility, acceptability, and preliminary estimates of a multimodal AKTIVER Heart Delirium Prevention Program in patients indicated for cardiac surgery: study protocol of a randomized controlled PREVENT POstoperative Delirium (PREVENT-POD) Pilot Study
Source: Pilot Feasibility Stud. 2026 Oct 5;12:142. doi: 10.1186/s40814-026-01923-6 (PMC13637325; doi:10.1186/s40814-026-01923-6)
Supplement: Supplementary file 5 [file 40814_2026_1923_MOESM5_ESM.pdf]

Statistical Analysis Plan

Version: 1.0 Date: 15.04.2026

Machbarkeit, Akzeptanz und vorläufige Wirksamkeit eines multimodalen AKTIVER Heart Delir-Präventionsprogramms bei Patient*innen mit Indikation zu einem herzchirurgischen Eingriff: Randomisiert kontrollierte PREVENT POstoperative Delirium (PREVENT-POD) Pilotstudie

PREVENT-POD

**Study Protocol**	2.0/04.10.2024
**EudraCT No.**	-
**Clinicaltrials.gov-No. / DRKS - No.**	DRKS00035091
**Internal Protocol ID No.**	2023-02947
**NCT-No.**	-
**SAP Version**	1.0/ 15.04.2026
**SAP Revisions**	
**Development Phase**	Pilot study
**Sponsor**	University Medical Center Göttingen<br>represented by the Head of Clinical trials unit UMG<br>Robert-Koch-Str. 40<br>37075 Göttingen, Germany
**Coordinating Investigator**	Dr. med. Monika Sadlonova<br>Klinik für Herz-, Thorax- und Gefäßchirurgie<br>Robert-Koch-Str. 40<br>37075 Göttingen, Germany

Approval of the Statistical Analysis Plan

Machbarkeit, Akzeptanz und vorläufige Wirksamkeit eines multimodalen AKTIVER Heart Delir-Präventionsprogramms bei Patient*innen mit Indikation zu einem herzchirurgischen Eingriff: Randomisiert kontrollierte PREVENT POstoperative Delirium (PREVENT-POD) Pilotstudie

EudraCT No.:

Protocol Version No: 2.0/04.10.2024

Coordinating Investigator	21.04.2026	
PD. Dr. Monika Sadlonova	Date	Signature
Responsible Trial Statistician	24.04.2026	
Dr. Thomas Asendorf	Date	Signature
Trial Statistician	24.04.2026	
Carlotta Derad	Date	Signature

Table of Contents

Statistical Analysis Plan 1
Approval of the Statistical Analysis Plan 2
Table of Contents 3
List of Figures 4
List of Tables 4
List of Abbreviations 5
1 Introduction 6
1.1 Background and Rationale 6
1.2 Objectives and Endpoints 6
1.3 Primary objective and endpoint 7
1.4 Secondary objectives and endpoints 8
1.5 Tertiary/other objectives and endpoints 8
2 Study methods 8
2.1 Trial design 8
2.2 Randomization 8
2.3 Sample Size 8
2.4 Framework 9
2.5 Statistical Interim Analyses and Stopping Guidance 9
2.6 Timing of the Final Analysis 9
2.7 Timing of Outcome Assessments 9
3 Statistical Principles 10
3.1 Confidence intervals and p-values 10
3.2 Adherence and protocol deviations 11
3.3 Analysis populations 11
4 Trial population 11
4.1 Screening data 11
4.2 Eligibility 11
4.3 Recruitment 11
4.4 Withdrawal/follow-up 11
4.5 Baseline patient characteristics 12
5 Analysis 12
5.1 Outcome definitions 12
5.2 Analysis methods 15
5.3 Missing data 17
5.4 Additional analyses 17
5.5 Harms 17
5.6 Statistical software 17
5.7 References 18

List of Figures

No table of figures entries found.

List of Tables

Table 1 Objectives and related endpoints ... 6

List of Abbreviations

AE	Adverse Event
AKTIVER	Alltags- und Kognitionstraining und Interdisziplinarität Verbessert Ergebnis und mindert das Risiko
CAM	Confusion Assessment Method
CFS	Clinical Frailty Scale
CI	Confidence Interval
CONSORT	Consolidated Statement of Reporting Trials
CSHA	Canadian Study of Health and Aging
DRKS	Deutsches Register Klinische Studien
DRS-R-98	Delirium Rating Scale-R-98
DSM-5	Diagnostic and Statistical Manual of Mental Disorders, Fifth Edition
eCRF	electronic Case Report Form
GCP	Good Clinical Practice
HADS	Hospital Anxiety and Depression Scale
IPQ	Illness Perception Questionnaire
IES-R	Impact of Event Scale-revised
MNA	Mini Nutritional Assessment
MoCA	Montreal Cognitive Assessment
mod.	modified
POD	Postoperative Delirium
PREVENT-POD	PREVENT POstoperative Delirium in Cardiac Surgery
PSQI	Pittsburgh Sleep Quality Index
SAE	Severe Adverse Event
SARC-F	Strength, Assistance with walking, Rise from a chair, Climb stairs and Falls
SDV	Source Data Verification
SMI	Self-reported subjective memory impairment
SOPs	Standard Operating Procedures
SWE	General Self-Efficacy (Selbstwirksamkeitserwartung)
TEX-Q	Treatment expectations questionnaire short form
UMG	Universitätsmedizin Göttingen

1 Introduction

This document has been written based on information contained in the study protocol version 2.0 from 04.10.2024.

1.1 Background and Rationale

Postoperative delirium (POD) is an acute and highly prevalent complication after cardiac surgery that can be associated with increased morbidity and mortality. One of the most effective methods of POD management is POD prevention. The evidence-based AKTIVER POD prevention program significantly reduced POD incidence and days with POD in orthopaedic and visceral surgery patients, but not in cardiac surgery patients. The lack of efficacy of the AKTIVER program in cardiac surgery demonstrated the need for a modification of the POD prevention program to address specific aspects of cardiac surgery patients. Therefore, the interdisciplinary multimodal and modified AKTIVER Heart POD prevention program was developed based on our own preliminary work and current research data.

The randomized controlled feasibility and pilot study PREVENT POstoperative Delirium in Cardiac Surgery (PREVENT-POD) will evaluate the acceptance, feasibility (study design and intervention) and preliminary efficacy of the AKTIVER Heart program in patients with an indication for elective cardiac surgery.

1.2 Objectives and Endpoints

Objectives of PREVENT-POD are

1. The multimodal, non-pharmacological AKTIVER Heart POD prevention program for cardiac surgery patients aged ≥65 years is feasible and well accepted by patients with an indication for elective cardiac surgery.
2. The study design of PREVENT-POD is feasible.
3. Preliminary efficacy of the AKTIVER Heart POD prevention program compared to the control group.

Primary and secondary endpoints are listed in Table 1.

Table 1 Objectives and related endpoints

	Objective	Endpoint
**Primary**	To show that the AKTIVER Heart POD prevention program is feasible and well accepted by the patients.	Feasibility and Acceptance of the AKTIVER Heart POD prevention program.
	To show that the study design of PREVENT-POD is feasible.	Feasibility of the PREVENT-POD study design.
**Secondary**	To evaluate whether the AKTIVER Heart intervention leads to a reduction in POD incidence compared to the TAU+.	Difference in POD incidence assessed by the CAM-ICU / 3D-CAM / 4-AT

	Objective	Endpoint
	To evaluate whether the AKTIVER Heart intervention leads to a preliminary reduction in POD severity and duration compared to TAU+.	Difference in Severity assessed by CAM-S and DRS-R-98.<br>Difference in duration, defined as sum of days with at least one positive POD-screening
	To determine the preliminary reduction in ICU and overall hospital length of stay.	Difference in ICU and overall hospital length of stay.
	To determine the preliminary improvement on cognition.	Change MoCA and 6-CIT from baseline to discharge and to 3mo-FU.
	To determine the preliminary improvement on frailty and nutrition.	Change in CSHA, Hand grip strength and MNA-SF from baseline to discharge and to 3mo-FU.
**Other (exploratory)**	To determine the preliminary improvement on depression and anxiety, sleep quality and PTBS.	Difference in depression and anxiety (HADS) and sleep quality (PSQI) from baseline to 3mo-FU.<br>Difference in IES-R at 3mo-FU.
	To assess illness perception and treatment expectations.	Difference in treatment expectations (TEX-Q) at baseline.<br>Difference in illness perception (IPQ) at 3mo-FU.
	To determine the safety.	Report of peri-/postoperative complications and adverse events (AE/SAE).
	To determine the impact on mortality.	Difference in all-cause mortality within 30 and 90 days after surgery.

1.3 Primary objective and endpoint

Feasibility of the AKTIVER Heart POD prevention program is assumed if 90% of the patients in the AKTIVER Heart intervention group (N = 36) have received

- preoperative information on perioperative procedures, postoperative delirium and preoperative somatic (optimization by the cardiac surgeons) and
- psychological preparation (approx. 10-14 days before the planned procedure) and
- at least 1 module per day after extubation for a period of five postoperative days.

Acceptance of the AKTIVER Heart POD prevention program is assumed if the acceptance of the intervention is rated by the AKTIVER patients with an average of ≥ 7.0 points on a scale of 0 (not at all acceptable) to 10 (very acceptable).

Feasibility of the study design is assumed if an average of one patient per week is randomized into the study (36 patients in 36 weeks, 24 in the AKTIVER group and 12 in the TAU+ group) and at least 75% of the randomized patients complete all assessments at the 3-month follow-up.

1.4 Secondary objectives and endpoints

Focus of the secondary endpoints is the POD incidence, duration (in days) and severity measured by CAM-ICU, 3D-CAM, 4-AT, CAM-S and DRS-R-98. Additionally, difference in ICU (in hours) and overall hospital length of stay (in days), in cognition measured by MoCA and 6-CIT as well as in frailty (CSHA, hand grip strength) and nutrition (MNA) will be analysed.

1.5 Other objectives and endpoints

Other endpoints will be analysed exploratory. For further details on the definition of the endpoints, see Section 5.1.

2 Study methods

2.1 Trial design

PREVENT-POD is a national, single-center, controlled randomized feasibility and pilot study. Patients in the intervention group (AKTIVER) will receive the multimodal, non-drug ACTIVE Heart Delirium prevention program. In addition to the standard treatment, patients in the control group (TAU+) receive information about the perioperative course and postoperative delirium (identical to the information given to the intervention group).

2.2 Randomization

Patients are randomized in a 2:1 ratio to either AKTIVER or TAU+ group using the randomization module of the REDCap Database. A biostatistician of the Department of Medical Statistics generated the allocation sequence. Randomization is based on permuted blocks of varying length.

2.3 Sample Size

The sample size estimation is based on the primary outcome measure and relevant effect size. The calculation was performed using nQuery Version 9.2.1.0.

Feasibility of the study intervention

Feasibility of the intervention is considered achieved if 90 % of patients in the AKTIVER Heart intervention group (N=36) receive preoperative education about the perioperative process, POD, and preoperative somatic and psychological preparation, as well as at least one AKTIVER Heart module per day after extubation for five days. As a minimal requirement, we aim to show superiority over 50% completion rate, therefore a one-sided binomial model will be applied at a significance level of 2.5 %: H0: $p \leq 0.5$ vs. H1: $p > 0.5$. Assuming a conservative 5-day completion rate of 75 % and a sample size of 40 patients, the power of the underlying test is 90%. The completion rate can be estimated with an accuracy of ± 13.6

Feasibility of the study design

Feasibility of the study design is considered achieved if, on average, one patient is randomized per week (36 patients over 36 weeks, with 24 in the AKTIVER Heart intervention group and 12 in

the TAU+ group – reflecting the 2:1 allocation ratio), and at least 75 % of the randomized patients complete all assessments by the 3-month follow-up. Assuming that the true proportion is actually 75% and a 2:1 allocation ratio (40 AKTIVER and 20 TAU+ patients); the proportion of patients who complete all 3-month assessments can be estimated with a precision of ± 11 %.

Acceptance of AKTIVER Heart intervention

The acceptance of the AKTIVER-Heart intervention is considered achieved if the average patient rating of acceptability - assessed on a scale of 0 (not acceptable at all) to 10 (highly acceptable) - is at least 7.0. Assuming a standard deviation of 3, this mean can be estimated in the intervention group with an accuracy of ±0.93 (95% CI width is 1.86).

2.4 Framework

If not stated otherwise, endpoints are tested for superiority of AKTIVER over TAU+.

2.5 Statistical Interim Analyses and Stopping Guidance

No interim analyses are planned. As no significant harms are to be expected, no stopping guidelines are planned.

2.6 Timing of the Final Analysis

The final analysis will take place when all outcomes have been collected and the database is locked.

2.7 Timing of Outcome Assessments

Four assessment times are planned for the patients as shown in the table below.

Table 2 Timing and instruments for outcome assessment of the PREVENT-POD study.

Stage	Preoperative assessment		Elective cardiac surgery	Postoperative assessment		
Time	Screening	Baseline/ Randomisation		Postoperative Day 1-5[1]	1-2 days before discharge	3 months after surgery
		T0/T1		T2	T3	T4
Inclusion / exclusion	x					
Information, consent and registration		x				
DRSQ		x				
ACB Score		x		x	x	x
EuroSCORE II and ASA		x				
Routine Examination		x		x	x	

[1] Longer in case of POD

Clinical laboratory parameters		x		x	x	x
Sociodemographics, medical history, medication		x				x
MoCA		x			x	x
SMI		x			x	x
HeartQoL		x				x
EQ-5D-5L		x				x
PSQI		x			x	x
HADS		x				x
IES-R						x
SWE		x			x	x
IPQ-B		x				x
TEX-Q		x				
CSHA-Frailty		x			x	x
SARC-F		x			x	x
Hand grip strength		x		x	x	x
MNA-SF		x		x		x
CAM-ICU / 3D-CAM / 4-AT		x		x		
CAM-S / DRS-R-98				x		
Duration and type of POD[2]				x		
Use of antipsychotics				x	x	
Use of fixation measures				x	x	
Peri- and postoperative complications					x	x
Length of hospital stay					x	
Feasibility of AKTIVER Heart					x	
Acceptance of AKTIVER Heart					x	
Qualitative interview on AKTIVER Heart					x	
Surgical protocol					x	
Institutionalization / need for care						x
30- and 90-day-mortality						x
AE / SAE				x	x	x

3 Statistical Principles

3.1 Confidence intervals and p-values

Unless stated otherwise, tests will be performed two-sided on a significance level of 5% and confidence intervals (CI) will be reported with 95% confidence level.

[2] If present

3.2 Protocol deviations

Patients who did not received surgery are excluded from the analysis.

3.3 Analysis populations

The primary analysis of the trial will follow the Intention-to-treat (ITT) principle. The ITT population consists of all randomized patients. Patients will be analysed according to the randomized treatment.

4 Trial population

4.1 Screening data

Screening data will be reported and described within a CONSORT flowchart.

4.2 Eligibility

Inclusion and exclusion criteria are listed in Table 3.

Table 3 Inclusion and exclusion criteria

Inclusion criteria	Exclusion criteria
1. Patients ≥ 65 years old<br>2. Elective cardiac surgery (duration of surgery > 60 minutes, with or without heart-lung machine, with or without sternotomy)<br>3. Ability to give consent<br>4. Willingness to participate voluntarily after informed consent with signed consent form<br>5. Ability to speak, read and understand German	1. Lack of capacity to consent<br>2. Severe dementia, severe mental disorders, acute delirium<br>3. Diagnosis of acute alcohol or drug abuse<br>4. Unstable angina pectoris, STEMI or NSTEMI within the last 2 weeks<br>5. Heart failure, NYHA IV<br>6. Severe cardiac arrhythmia refractory to therapy<br>7. Advanced (metastasized) oncological disease<br>8. Participation in another intervention study (exception participation in registry studies)

4.3 Recruitment

Recruitment numbers will be reported and described within a CONSORT flow diagram.

4.4 Withdrawal/follow-up

Any trial patient may discontinue treatment or withdraw consent to participate in the trial at any time without giving a reason.

4.5 Baseline patient characteristics

Baseline characteristics will be summarized using descriptive statistics (e.g. mean, standard deviation, median, interquartile range for continuous variables and frequencies (percentages) for categorical variables) and appropriate graphical methods (e.g. boxplots, histograms, barplots) depending on the data type.

5 Analysis

5.1 Outcome definitions

A full list of outcomes and their timing is described in Section 2.7.

Feasibility and acceptance of AKTIVER Heart POD prevention program

One to two days before discharge feasibility and acceptance will be assessed in the AKTIVER group patients. Feasibility will be assessed by evaluating whether patients received preoperative education on perioperative procedures and POD as well as perioperative somatic and psychological preparation and at least one AKTIVER Heart module per day. Acceptance will be assessed using a structured interview and the evaluation of various aspects on numerical scales ranging from 0 to 10.

Additionally, study surgeons will compile a standardized surgical protocol and a record of intra-, peri-, and postoperative complications from the electronic patient record for both groups. Length of stay in the intensive care unit, as well as the total treatment duration (post-discharge), will also be documented.

Feasibility of the study design

Feasibility of the study design is considered achieved, if, on average, one patient is randomized per week (e.g., 36 patients over 36 weeks, with 24 in the intervention group and 12 in the control group – reflecting a 2:1 randomization), and at least 75% of the randomized patients complete all assessments at 3-month follow-up.

DRSQ

The individual delirium risk of the patients is assessed using the Delirium Risk Screening Questionnaire (DRSQ), which was originally developed in the PAWEL study (Sánchez et al.

2019). The DRSQ consists of three parts accounting for potential delirium risks:

1. The Geri-check incudes five items mobility, independence in activities of daily living, cognition, mental health, and previous hospitalization. If at least two items are positive, the patient is classified as geriatric patient.
2. The 6-Item Cognitive Impairment Test (6-CIT) assesses orientation, short-term memory and attention. The 6-CIT score ranges from 0 to 29 points (0-7 normal cognition, 8-9 mild cognitive impairment, 10-28 significant cognitive decline).
3. 19 items on general patient information, including Age <70 years, laboratory parameters, ASA score ≥ 3, medications per day > 6, ACB score ≥ 1, self-reported subjective memory impairment (SMI) and hand grip strength.

Hand grip strength is measured using the Jamar® Hydraulic Hand Dynamometer. A sum score of <2 requires no further action, a score of 2–4 indicates the need for further delirium risk assessment, and a score of ≥4 warrants the implementation of preventive measures.

ACB

The Anticholinergic Cognitive Burden (ACB) score assesses anticholinergic load and is based on a list of medications commonly used in Germany. A medication with no anticholinergic effect has an ACB score of 0, a mild anticholinergic effect is assigned a score of 1, a moderate effect a score of 2, and a strong effect a score of 3. The total ACB score is the sum of individual medication scores. If the total score is ≥3, it a reduction of the burden is recommended.

CFS

Frailty is measured by the German version of the Clinical Frailty Scale (CFS), which comprises nine categories (1 = very fit, 9 = extremely ill). A score of 5 or higher indicates frailty.

SARC-F

The "Strength, Assistance with walking, Rise from a chair, Climb stairs and Falls" (SARC-F) questionnaire is used to identify patients with sarcopenia. Each of the five questions is rated on a scale from 0 to 2, and the scored items are summed to calculate the total score. The maximum total score is 10, with a score of ≥4 indicating sarcopenia.

MNA-SF

The risk of malnutrition is assessed using the Mini Nutritional Assessment - Short Form (MNA-SF). The questionnaire evaluates food intake and weight loss over the last three months, mobility, acute illness and psychological stress, neuropsychological issues, and body weight. For each category, up to three points can be scored. The scored items are summed to calculate the total score. The higher the total score, the lower the risk of malnutrition (12-14 normal nutritional status, 8-11 risk of malnutrition, 0-7 presence of malnutrition).

MoCA

Cognitive function is assessed using the Montreal Cognitive Assessment (MoCA), a 30-item test covering multiple cognitive domains. The maximum score is 30, with a total score of ≤26 indicating mild cognitive impairment. For follow-up assessments conducted via telephone, the MoCA-22 is assessed. In both versions, a single missing value invalidates the score. Transformation of MoCA-22 scores to the 30-point scale will be performed using the equipercentile method (Katz et al. 2020).

HeartQoL

Health-related quality of life is assessed using the Heart Quality of Life (HeartQoL) questionnaire, a 14-item self-report instrument with a physical and an emotional subscale for patients with ischemic heart disease. Each item is scored from 0 (“A lot”) to 3 (“No”). Subscale scores are calculated by determining the mean of the respective items. The physical subscale consists of 10 items (up to 5 missing items allowed) and the emotional subscale of four items (up to 2 missing items allowed). The total HeartQoL score ranges from 0 to 3, with a maximum of 7 missing items permitted.

EQ-5D-5L

Overall quality of life is measured using the five-dimensional EuroQoL questionnaire (EQ-5D-5L). Each of the five dimensions (mobility, self-care, usual activities, pain/discomfort, and anxiety/depression) is rated on a 5-point scale with higher values indicating severe problems. This

results in a 5-digit code representing one of 3125 possible health states. These health states are transformed into a quality-of-life index using the German validation set. If any dimension is missing, the index cannot be calculated. Additionally, patients assess their overall health status on a visual analog scale (EQ-VAS; range 0–100).

PSQI

The Pittsburgh Sleep Quality Index (PSQI) is used for the assessment of sleep quality in clinical settings. The seven components (subjective sleep quality, sleep latency, sleep duration, sleep efficiency, sleep disturbances, use of sleep medication, and daytime dysfunction) evaluate subjective sleep quality over the past four weeks. Scores for each question range from 0 to 3.The total score, defined as sum of the scored items, ranges from 0 to 21, with a score >5 indicating impaired sleep quality.

HADS

The Hospital Anxiety and Depression Scale (HADS) is used to assess symptoms of anxiety and depression. It consists of 14 items, each rated on a four-point Likert scale. The scale comprises two subscales, one for anxiety and one for depression, each containing seven items and yielding a maximum score of 21. If necessary, one missing item per subscale can be imputed by the rounded mean of the remaining six items. The HADS total score ranges between 0 and 42 points, with higher scores indicating greater severity.

SWE

Self-efficacy is assessed using the General Self-Efficacy Scale (SWE). The scale consists of 10 self-reported items evaluating the individual’s perceived effectiveness in finding solutions and managing difficulties and challenges. Each item is scored on a scale from 1 to 4. The total score is the sum of the ten rated items (range 10-40).

TEX-Q

Patient expectations regarding cardiac surgery were measured using the 15-item Treatment Expectation Questionnaire (TEX-Q). Items are rated on an 11-point scale (0-10). The overall TEX-Q score is calculated as the mean of all items after reversing the scores for items 7 through 11. Subscale scores, representing Treatment Benefit (Items 1-3), Positive Impact (Items 4-6), Adverse Events (Items 7-9), Process (Items 12-13), and Behavioral Control (Items 14-15), are calculated as the mean of the respective items.

IPQ-B

Disease-related expectations were assessed using the short version of the Illness Perception Questionnaire (IPQ-B). This 8-item questionnaire uses a 0-10 scale. The IPQ-B will be analyzed at both, the single-item level and the total score level. The total score is the sum of all items (range 0-80) with higher scores indicating a worse illness perception. Items 3 (personal control), 4 (treatment control), and 7 (understanding) required reverse scoring. Furthermore, the patients are asked to specify the most important subjective reasons for the perceived cause of the disease.

Delirium

It is preoperatively assessed whether delirium is already present in the patients. Over the first five postoperative days, patients are screened for delirium after extubation twice daily using the CAM-ICU and 4-AT in the intensive care unit and the 3D-CAM and 4-AT in the general ward. In case of POD, the patient is screened until the delirium subsides and the duration of POD will be documented. Since POD symptoms often manifest at night, a chart review will also be conducted. Additionally, electronic documentation will be reviewed, focusing on:

a. Delirium symptoms during the night,

b. Use of psychotropic drugs or dexmedetomidine, with a focus on antipsychotics and

c. use of restraint measures.

Delirium severity is assessed twice daily using the I-CAM-S and the Delirium Rating Scale-Revised-98 (DRS-R-98).

3D-CAM

The 3D-CAM is used to evaluate delirium incidence and consists of three assessment categories (patient questions, observer ratings and two optional items). In the first category, seven questions assess thinking and memory, and the patient provides a subjective assessment of their current mental state, orientation, and presence of hallucinations. Answers are marked as true or false, or yes or no for subjective questions. 10 observational criteria are answered yes or no. Each question and task in the 3D-CAM corresponds to characteristics 1, 2, 3 or 4. If characteristics 1 and 2 and either 3 or 4 are present, delirium is indicated. The two optional items on acute change only need to be addressed if characteristic 1 is absent but characteristics 2 and either 3 or 4 are present.

4-AT

The 4-AT is used to evaluate delirium incidence and consists of four items (level of alertness, Abbreviated Mental Test-4, evaluation of attention by backwards reciting the months of the year and acute change in mental state or fluctuation of symptoms). The maximum of the score is 12 points (0 points no delirium/cognitive impairment; 1-3 points possible cognitive impairment, ≥ 4 points possible delirium).

CAM-S

The CAM-Short Form (CAM-S) is a brief questionnaire that assesses the severity of POD using four features (acute change or fluctuation, attentional disorders, disorganized thinking and altered state of consciousness). Each feature is scored as absent (0), mild (1) or severe (2), with the exception of fluctuation (absent = 0, present = 1). The total score ranges from 0 (none) to 7 (most severe).

DRS-R-98

The DRS-R-98 comprises a total of 16 items, including 13 severity items and 3 diagnostic items for delirium. The severity items are rated on a scale from 0 to 3, while the diagnostic items are rated on a scale of either 0–2 or 0–3. A score of 0 indicates the absence of delirium. The maximum possible score for the severity items is 39, and the overall maximum score is 46 with higher scores indicating a more severe delirium.

5.2 Analysis methods

Primary endpoint

The primary endpoint will be analysed using descriptive statistics.

Feasibility of the intervention is measured by the proportion of patients in the AKTIVER Heart intervention group, who receive preoperative education about the perioperative process, POD, and preoperative somatic and psychological preparation, as well as at least one module per day after extubation for the duration of five days.

Feasibility of the study design is measured through the average number of patient randomized per week, and the proportion of randomized patients who completed all assessments at the 3-month follow-up.

Acceptance of the AKTIVER Heart intervention is measured by the average patient rating of acceptability, which is assessed on a scale of 0 (not acceptable at all) to 10 (highly acceptable). The statement of main concern is “I was able to accept the therapeutic measures of the AKTIVER Heart delirium prevention program well during my inpatient stay” (“Die durgeführten therapeutischen Maßnahmen des AKTIVER Heart Delir-Präventionsprogramms konnte ich im Rahmen des stationären Aufenthaltes gut annehmen.”).

Secondary endpoints

For the secondary outcomes, the patient assessment results of the AKTIVER group will be compared with those of the TAU+ group.

For delirium incidence and other dichotomous endpoints, differences between the groups will be examined using logistic regression. Odds ratio (OR) will be reported along with 95% CIs.

```
library(gtsummary)

mod <- glm(pod ~ intervention, data = data, family = binomial)
tbl_regression(mod, exponentiate = TRUE)
```

Differences in continuous variables (delirium severity, MoCA, frailty, MNA-SF, HADS, PSQI, IES-R, IPQ and TEX-Q) are analyzed using generalized linear mixed models (GLMM). Marginal effects are calculated as estimated marginal means, adjusting for baseline values, and are compared between AKTIVER and TAU+ groups at different time points. Effect estimates (marginal differences) are reported with 95% CIs.

```
library(mmrm)
library(emmeans)

model <- mmrm(var ~ var.bl + group * time,
              covariance = cov_struct('us', 'time', subject = 'PatID'),
              data = data)
tmt.means <- emmeans(mod, ~ intervention * time)
tmt.means
contrast(tmt.means, method = "revpairwise", by = "time")
```

Differences in count data namely delirium duration, ICU and overall hospital length of stay are analyzed using either Poisson regression or negative binomial regression, depending on the observed overdispersion. Should there be a high number of zero-observations; zero-inflated models are considered alternatively. Group-specific rates are reported with 95% CIs.

```
library(MASS)
library(emmeans)


# Negative binomial Regression
mod <- glm.nb(count ~ intervention, data = data)
tmt.means <- emmeans(mod, ~ intervention , type = "response")
tmt.means
contrast(tmt.means, method = "revpairwise")
```

The time-to-event variables, e.g. 30- and 90-day mortality, is analyzed using Cox proportional hazard models with treatment group, age and type of surgery as factors. Hazard ratios (HR) will be reported with 95% CIs. Kaplan-Meier curves and/or adjusted survival curves for Cox PH models will be used to illustrate the treatment effect. The proportional hazards assumption will be checked visually by graphical diagnostics including scaled Schoenfeld residuals.

```
library(survival)
library(gtsummary)

SurvObj <- Surv(FU, prim.end, data = data)
cox.regr <- coxph(SurvObj ~ group, data = data)
tbl_regression(cox.regr, exponentiate = TRUE)
# check ph assumption
test.ph <- cox.zph(cox.regr)
ggcoxzph(test.ph)
# plot KM curves
plot(survfit(SurvObj ~ group, data = data, conf.type = "log-log"))
```

P-values corresponding to the null hypothesis of no intervention effect will be reported for descriptively purposes only.

5.3 Missing data

In case of missing data, all patients will be analysed following the intention-to-treat principle. No imputation methods are planned, as the amount of missing data is expected to be minimal.

5.4 Additional analyses

The dataset will be provided to the study team for further analysis. Further analyses will be performed in doctoral theses.

Furthermore, patients with POD will be considered as a separate subgroup for analyses of long-term outcomes such as quality of life, with individual patient data displayed in a line-listing format.

5.5 Harms

There is no legal obligation to report adverse events to the competent authority as no drug or medical device are tested in PREVENT-POD. A selection of adverse events will be recorded to monitor certain safety aspects of the study intervention and to evaluate the study outcome.

5.6 Statistical software

All analyses will be performed in the current version of R. R packages are used in their current version and will be reported in the statistical report. For the EQ-5D-5L index value calculation, the R package *eq5d* will be used (Morton F, Nijjar JS 2024).

5.7 References

1. Sánchez, A., Thomas, C., Deeken, F., Wagner, S., Klöppel, S., Kentischer, F., von Arnim, C. A. F., Denkinger, M., Conzelmann, L. O., Biermann-Stallwitz, J., Joos, S., Sturm, H., Metz, B., Auer, R., Skrobik, Y., Eschweiler, G. W., Rapp, M. A., & PAWEL Study group (2019). Patient safety, cost-effectiveness, and quality of life: reduction of delirium risk and postoperative cognitive dysfunction after elective procedures in older adults-study protocol for a stepped-wedge cluster randomized trial (PAWEL Study). Trials, 20(1), 71. https://doi.org/10.1186/s13063-018-3148-8
2. Katz MJ, Wang C, Nester CO, et al. T-MoCA: A valid phone screen for cognitive impairment in diverse community-samples. Alzheimer's Dement. 2021; 13:e12144. https://doi.org/10.1002/dad2.12144
3. Morton F, Nijjar JS (2024). eq5d: Methods for Analysing 'EQ-5D' Data and Calculating 'EQ-5D' Index Scores. R package version 0.15.3, https://CRAN.R-project.org/package=eq5d

See study protocol section 15
